# Development and validation of a postpartum hemorrhage risk prediction model for advanced maternal age women undergoing vaginal delivery: a comparison of multiple machine learning algorithms

**DOI:** 10.3389/fmed.2026.1898119

**Published:** 2026-09-04

**Authors:** Yun Zhou, Xiao Yao, Jue Zhou, Yaoxiang Duan, Yi Yu, Xueer Ma

**Affiliations:** Shanghai Key Laboratory of Maternal Fetal Medicine, Shanghai Institute of Maternal Fetal Medicine and Gynecologic Oncology, Shanghai First Maternity and Infant Hospital, School of Medicine, Tongji University, Shanghai, China

**Keywords:** advanced maternal age, algorithm comparison, machine learning, postpartum hemorrhage, risk prediction model

## Abstract

**Background:**

Postpartum hemorrhage (PPH) is the leading cause of maternal mortality in women of advanced maternal age (AMA). Accurate antenatal prediction remains challenging. This study aimed to develop and compare the performance of multiple machine learning (ML) algorithms for predicting PPH risk following vaginal delivery in AMA parturients.

**Methods:**

A retrospective cohort study was conducted using data from 5,369 women who delivered vaginally at Shanghai First Maternity and Infant Hospital. LASSO regression was used to identify key predictors. Seven machine learning algorithms—logistic regression (LR), decision tree (DT), random forest (RF), XGBoost, LightGBM, support vector machine (SVM), and artificial neural network (ANN)—were employed to build prediction models. Model performance was evaluated on an independent validation set using the area under the receiver operating characteristic curve (AUC), sensitivity, specificity, and F1 score. The optimal model was selected based on a composite criterion prioritizing the highest sensitivity and best F1 score, while requiring a competitive AUC.

**Results:**

Eight predictors were selected for model construction. Among the seven models, the SVM model achieved the highest sensitivity (0.482) and the highest F1 score (0.504), and was therefore selected as the clinically preferred model, while maintaining a competitive AUC of 0.782 (95% *CI*: 0.758–0.806). SVM achieved a favorable balance between sensitivity and specificity, indicating better clinical utility for early screening and risk stratification of postpartum hemorrhage.

**Conclusion:**

Among seven machine learning models, the SVM model was selected as the clinically preferred predictor due to its highest sensitivity and F1-score. Its good discriminative ability and balanced performance make it suitable for clinical application as a screening tool. The integration of SHAP analysis can further enhance model interpretability and facilitate individualized risk assessment.

## 1 Introduction

Postpartum hemorrhage (PPH) is a common obstetric complication that seriously endangers the health of both mothers and newborns (1). It is recognized as the leading cause of maternal mortality worldwide, accounting for 27.1% of maternal deaths (2). Related studies have indicated a positive linear association between maternal age and postpartum hemorrhage. With respect to maternal age, it is currently believed that the incidence of PPH is significantly greater in subjects aged 35 years and older (MD = 1.15, 95% *CI*: 1.03–1.27) (3). In 1958, the International Federation of Gynecology and Obstetrics (FIGO) defined pregnancies in women aged 35 years or older as advanced maternal age (AMA) pregnancies, and the women in this category are referred to as women of AMA (4). The trend of delayed marriage and childbearing, the rapid development of assisted reproductive technology, and the increasing rate of divorce and remarriage have contributed to a global increase in the proportion of AMA pregnancies (5). Women of AMA, as a unique demographic, experience some degree of decline in fertility and physiological functions because of aging. Advanced age can increase the likelihood of PPH and increase the risk of adverse outcomes (6, 7).

Clinically, it is believed that adverse outcomes caused by PPH are preventable, and accurate risk assessment serves as a crucial starting point for PPH prevention and management decisions, which helps reduce the incidence of PPH (8). Therefore, identifying the risk factors for PPH in AMA pregnancies is beneficial for identifying high-risk parturients. Currently, effective and timely bleeding assessment for high-risk parturients remains challenging (9, 10).

However, machine learning (ML) can extract key features from vast datasets to construct predictive models, thereby more accurately identifying high-risk individuals and enabling early intervention (11). In this study, the clinical records of 5,369 women of AMA who delivered at our hospital from December 2022 to December 2024 were retrospectively analyzed, and a risk prediction model for PPH following vaginal delivery in women of AMA was constructed using machine learning methods. We analyzed the data to identify key factors closely associated with the risk of PPH after vaginal delivery in women of AMA and compared the predictive performance of different machine learning models (6).

The primary aim of this study was to develop and internally validate a prediction model using multiple ML algorithms, thereby providing a reliable tool for more accurately assessing PPH risk in this population to inform clinical decision-making. Identifying risk factors for PPH in women of AMA and constructing a clinical predictive model can optimize outcomes for these women and prevent excessive blood loss in high-risk pregnant women (12).

## 2 Materials and methods

### 2.1 Study population

This investigation includes pregnant women of AMA who received antenatal care and underwent vaginal delivery at our hospital between December 2022 and December 2024.

### 2.2 Inclusion criteria

Based on the 2016 Guidelines for the Prevention and Management of Postpartum Hemorrhage, PPH was defined as a blood loss of ≥500 mL within 24 h after childbirth via vaginal delivery. All patients were aged ≥35 years and had complete clinical data.

### 2.3 Exclusion criteria

Patients with severe hepatic or renal insufficiency, hematopoietic system diseases, or a history of malignant tumors were excluded.

General patient information and related indicators were obtained from the electronic medical records in the Hospital Information System (HIS) of the department.

The following clinical thresholds were predefined based on institutional guidelines and international standards: prolonged second stage of labor was defined as >3 h for nulliparous women and >2 h for multiparous women (with epidural analgesia: >4 h and >3 h, respectively); prolonged third stage of labor was defined as >30 min; elevated D-dimer was defined as >0.5 mg/L fibrinogen equivalent units (FEU); macrosomia was defined as birth weight ≥4,000 g; gestational diabetes mellitus (GDM) was diagnosed according to the IADPSG criteria (75 g OGTT); premature rupture of membranes (PROM) was defined as rupture of membranes before the onset of labor; Group B Streptococcus (GBS) infection was defined as a positive screening culture during pregnancy.

### 2.4 Blood loss measurement and data management

In this study, blood loss was measured following the standard clinical practice in our institution, using quantitative methods: a combination of volumetric (using calibrated drapes) and gravimetric (weighing sanitary pads and gauze) techniques, supplemented by clinical estimation by midwives or obstetricians. The missing rate for all analyzed variables was below 2%; therefore, a complete-case analysis was applied for records with missing data.

### 2.5 Statistical analysis

All data cleaning and model building were performed using R version 4.4.2 and Python version 3.11.4. Descriptive statistics were first computed for baseline patient characteristics and relevant scores. Normality testing indicated that the continuous variables in this study were not normally distributed; however, given the large sample size, they are presented as mean ± standard deviation (SD), and group comparisons were performed using the Mann–Whitney *U*-test. Categorical variables are presented as frequencies, proportions, or percentages, and comparisons between or within groups were conducted using the chi-square test. The least absolute shrinkage and selection operator (LASSO) regularization technique was used to select risk factors strongly associated with the outcome, and these variables were then included in the models.

Seven machine learning algorithms were compared: logistic regression (a linear classifier), decision tree (a tree structure based on feature splitting), random forest (an ensemble of multiple decision trees that vote on the outcome), XGBoost (a gradient-boosted tree algorithm), LightGBM (a histogram-based gradient boosting method), support vector machine (which uses kernel functions to map features into a high-dimensional space and finds the optimal hyperplane), and artificial neural network (which simulates connections among neurons). All models were tuned using five-fold cross-validation on the training set. Model performance was evaluated on the validation set using the following primary metrics: area under the receiver operating characteristic curve (AUC) with its 95% confidence interval, precision, sensitivity, specificity, and F1 score. The optimal model was selected based on a composite criterion prioritizing high sensitivity and F1 score, while requiring a competitive AUC.

The SHAP (SHapley Additive exPlanations) method was applied to provide global and individual-level explanations of the best-performing model. Positive SHAP values indicate an increased risk of PPH, while negative values indicate a decreased risk. The visualizations included: (1) a global feature importance bar plot showing the mean absolute SHAP value for each feature (larger values indicate greater contribution); (2) a beeswarm plot displaying the distribution of SHAP values for each sample, with color representing feature value (red = high, blue = low); (3) a waterfall plot for a single sample, starting from the baseline expected value and adding or subtracting the contribution of each feature sequentially according to the magnitude of its SHAP value (red bars indicate risk factors, blue bars indicate protective factors, and bar length represents the absolute contribution); and (4) a force plot that presents the SHAP values for a single sample in a force-field format (red indicates positive contributions that increase risk, blue indicates negative contributions that decrease risk; a larger red area means higher predicted risk). Through these visualizations, both global and individual interpretations of the optimal model can be achieved, thereby enhancing model credibility and clinical acceptability.

Because the outcome of this study is whether PPH occurs (binary outcome) rather than time to occurrence, classification algorithms were used to build the models. A two-sided *P* value < 0.05 was considered statistically significant.

The entire dataset was randomly split into a training set (70%, *n* = 3,758) and an independent validation set (30%, *n* = 1,611), stratified by the outcome variable to preserve the PPH proportion. Feature selection using LASSO regression was performed on the training set only. Hyperparameter tuning for each machine learning algorithm was conducted via 5-fold cross-validation on the training set, with AUC as the scoring metric. The final models were then evaluated on the untouched validation set. All splits were performed with a fixed random seed (seed=123) to ensure reproducibility.

## 3 Results

### 3.1 Baseline characteristics of the maternal population

Baseline data of the postpartum hemorrhage (PPH) group and the non-PPH group are shown in Table 1.

**Table 1:** Comparison of baseline data between the two groups.

Variables	Total (*N* = 5,369)	Non-PPH (*n* = 4,109)	PPH (*n* = 1,260)	*P*
Hypertension in pregnancy, *n* (%)				0.452
No	4,122 (76.77)	3,165 (77.03)	957 (75.95)	
Yes	1,247 (23.23)	944 (22.97)	303 (24.05)	
Preterm birth, *n* (%)				0.100
No	4,797 (89.35)	3,655 (88.95)	1,142 (90.63)	
Yes	572 (10.65)	454 (11.05)	118 (9.37)	
Gestational diabetes mellitus, *n* (%)				<0.001
No	4,514 (84.08)	3,609 (87.83)	905 (71.83)	
Yes	855 (15.92)	500 (12.17)	355 (28.17)	
GBS infection, *n* (%)				<0.001
No	4,944 (92.08)	3,890 (94.67)	1,054 (83.65)	
Yes	425 (7.92)	219 (5.33)	206 (16.35)	
Nuchal cord, *n* (%)				0.047
No	4,880 (90.89)	3,753 (91.34)	1,127 (89.44)	
Yes	489 (9.11)	356 (8.66)	133 (10.56)	
Elevated D-dimer, *n* (%)				<0.001
No	3,839 (71.50)	2,763 (67.24)	1,076 (85.40)	
Yes	1,530 (28.50)	1,346 (32.76)	184 (14.60)	
PROM, *n* (%)				<0.001
No	4,356 (81.13)	3,402 (82.79)	954 (75.71)	
Yes	1,013 (18.87)	707 (17.21)	306 (24.29)	
Mode of delivery, *n* (%)				0.837
Spontaneous	4,586 (85.42)	3,507 (85.35)	1,079 (85.63)	
Forceps-assisted	783 (14.58)	602 (14.65)	181 (14.37)	
Macrosomia, *n* (%)				<0.001
No	4,953 (92.25)	3,870 (94.18)	1,083 (85.95)	
Yes	416 (7.75)	239 (5.82)	177 (14.05)	
Episiotomy, *n* (%)				0.263
No	4,501 (83.83)	3,458 (84.16)	1,043 (82.78)	
Yes	868 (16.17)	651 (15.84)	217 (17.22)	
First stage duration, *n* (%)				0.759
Normal	4,377 (81.52)	3,354 (81.63)	1,023 (81.19)	
Prolonged	992 (18.48)	755 (18.37)	237 (18.81)	
Second stage duration, *n* (%)				<0.001
Normal	4,350 (81.02)	3,530 (85.91)	820 (65.08)	
Prolonged	1,019 (18.98)	579 (14.09)	440 (34.92)	
Third stage duration, *n* (%)				<0.001
Normal	4,576 (85.23)	3,680 (89.56)	896 (71.11)	
Prolonged	793 (14.77)	429 (10.44)	364 (28.89)	
Prenatal checkup times, mean ± SD	11.76 ± 7.65	12.02 ± 7.84	10.92 ± 6.95	<0.001
Age (years), mean ± SD	35.54 ± 4.00	35.59 ± 3.88	35.37 ± 4.35	0.093
Gravida, mean ± SD	2.15 ± 1.16	2.24 ± 1.17	1.84 ± 1.10	<0.001
Para, mean ± SD	1.56 ± 0.61	1.63 ± 0.61	1.32 ± 0.53	<0.001

### 3.2 Variable selection for the PPH risk prediction model

**Figure 1:**
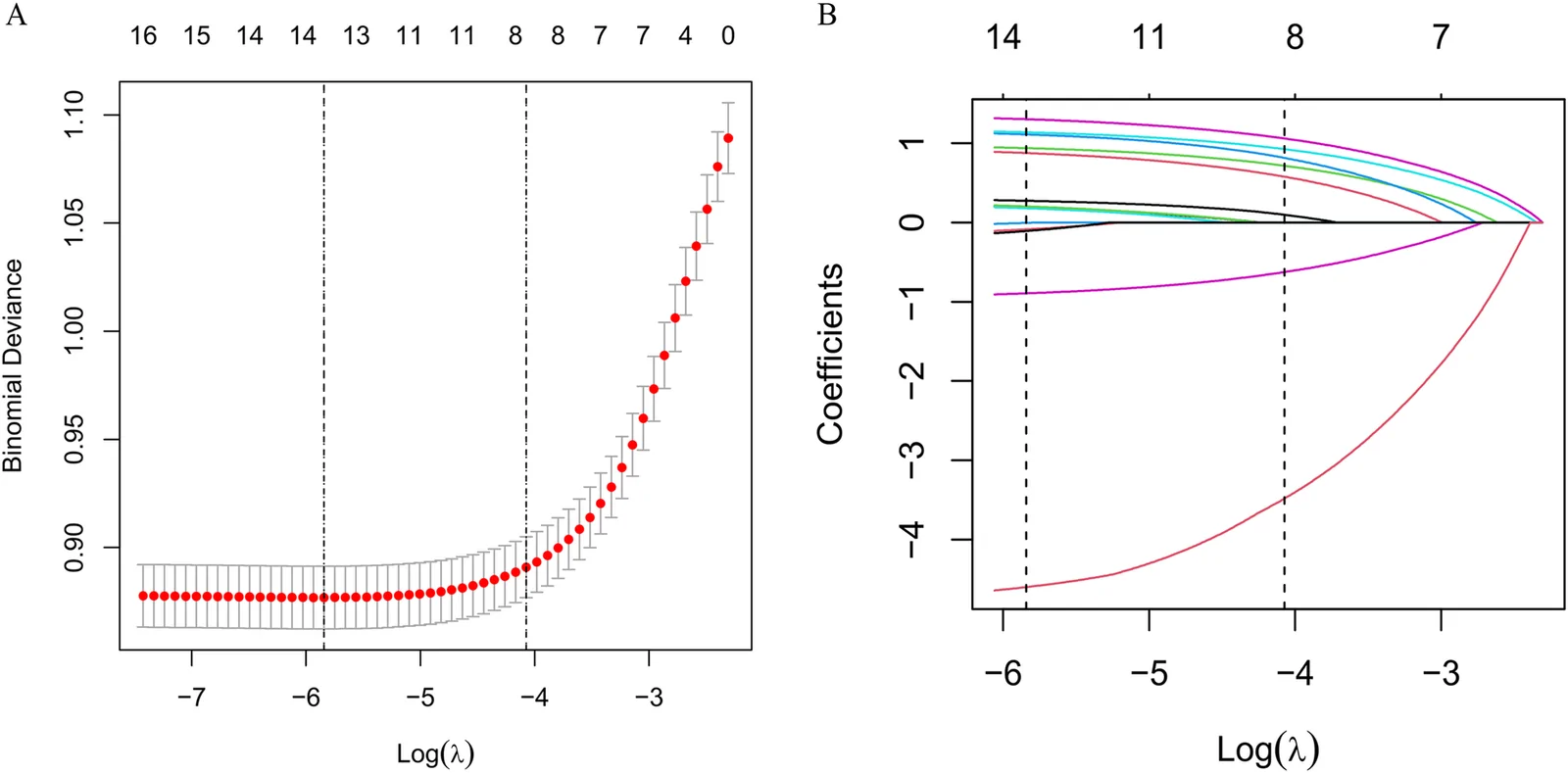
The results of the LASSO regression analysis. LASSO, Least Absolute Shrinkage and Selection Operator. **(A)** The coefficient trajectories of all 18 variables across different regularization parameters. **(B)** The cross-validation curves (5-fold cross-validation).

Among 18 variables, eight optimal predictors were identified using LASSO regression. As shown in Figure 1, these predictors were: parity, duration of the second stage of labor, duration of the third stage of labor, PROM, GBS infection, macrosomia, elevated D-dimer level, and GDM. A complete list of all candidate variables considered for LASSO regression, along with their types and selection status, is provided in Supplementary Table S1.

### 3.3 Comparison of model performance

The complete hyperparameter tuning details, including optimization strategy (GridSearchCV), search space, and final selected values for each model, are provided in Supplementary Data Sheet 1.

**Figure 2:**
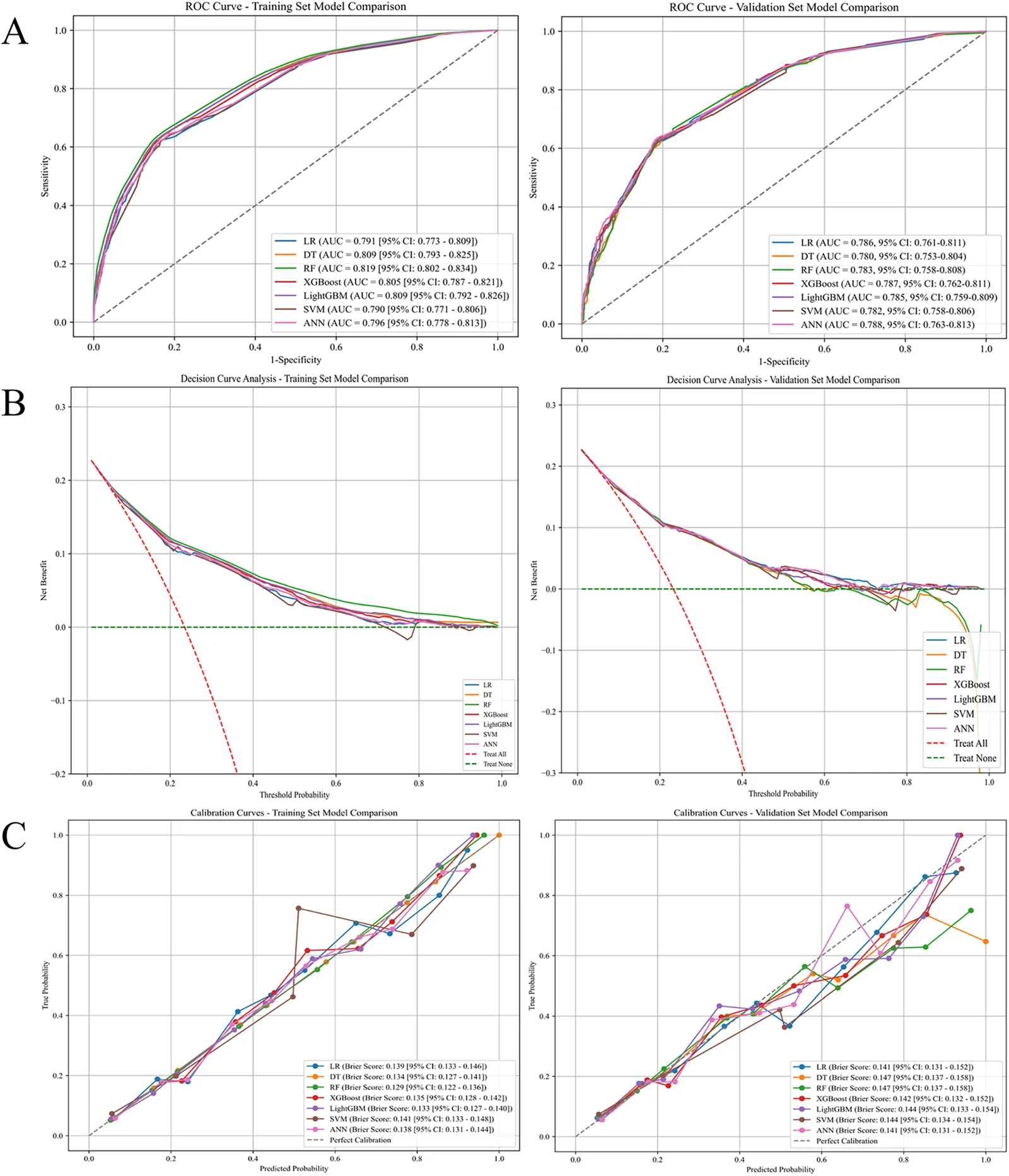
Prediction performance evaluation of seven machine learning models. **(A)** ROC curves of seven machine learning models in the training and validation sets. **(B)** Calibration curves of seven machine learning models in the training and validation sets. **(C)** Decision curves of seven machine learning models in the training and validation sets.

**Figure 3:**
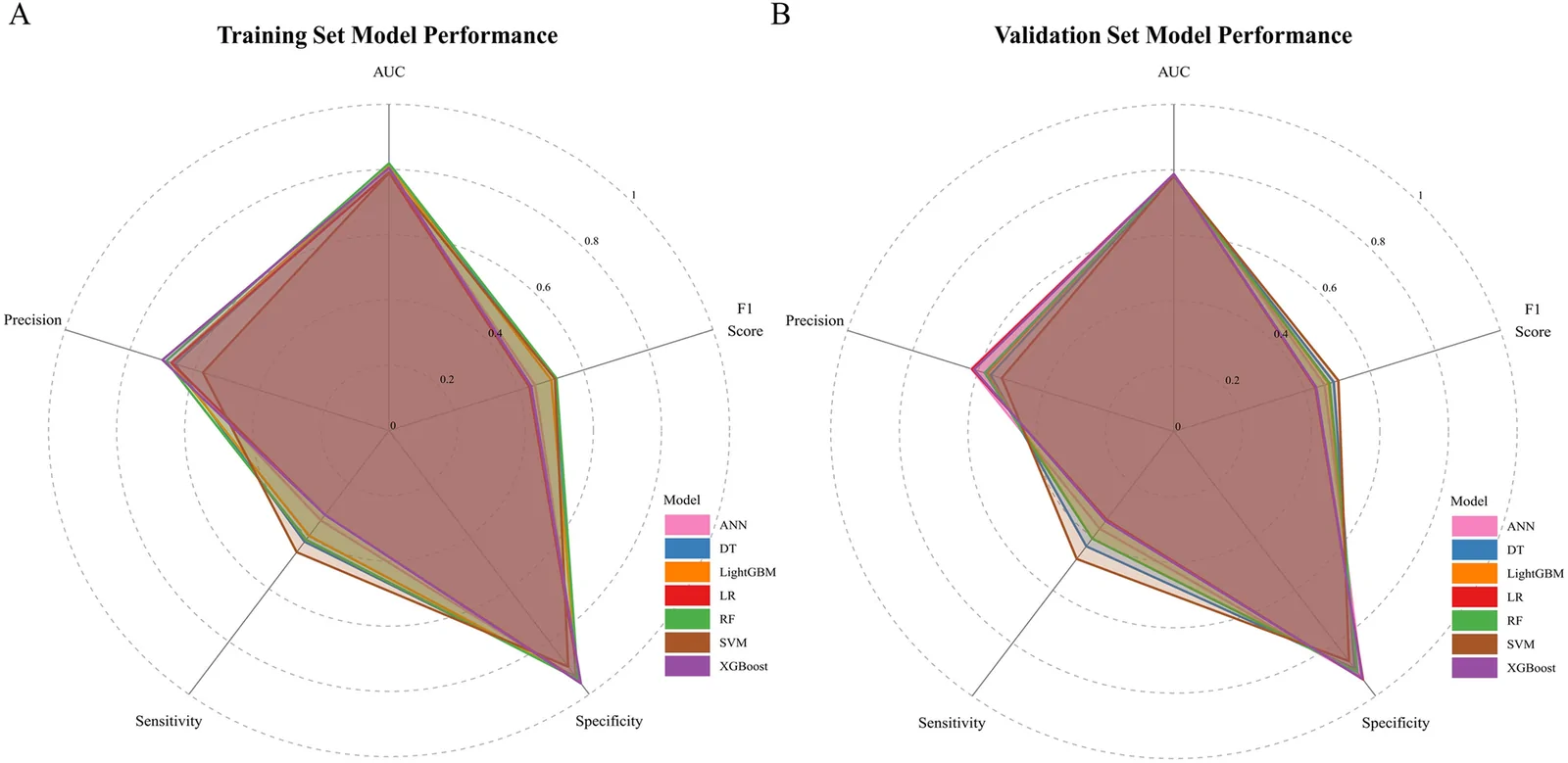
Radar charts of detailed performance metrics for seven machine learning models. **(A)** Detailed performance metrics of the models on the training set. **(B)** Detailed performance metrics of the models on the validation set.

As presented in Figure 2, the predictive performance of the seven machine learning models for PPH was evaluated using the training and validation sets. The AUC values of the models ranged from 0.780 to 0.788, with largely overlapping confidence intervals. Figure 3 shows that the SVM model achieved the highest sensitivity (0.482), the largest Youden index (0.349), and the best F1 score (0.504), indicating its optimal overall performance for identifying patients with PPH. The calibration curve of the SVM model demonstrated good agreement between predicted probabilities and observed outcomes. Decision curve analysis (DCA) further indicated that the SVM model provided greater net clinical benefit than the other models.

Pairwise comparisons using DeLong's test showed no statistically significant differences between the SVM model (AUC = 0.782, 95% *CI*: 0.758–0.806) and the other six models (all *P* > 0.05).

These findings confirm that all seven models exhibited comparable discriminative ability. The SVM model achieved a Brier score of 0.144, indicating good overall prediction accuracy. The calibration slope was 0.776 (95% *CI*: −0.184 to 0.873), suggesting modest miscalibration—a recognized limitation of SVM with Platt scaling.

### 3.4 SHAP interpretability analysis of the optimal model

**Figure 4:**
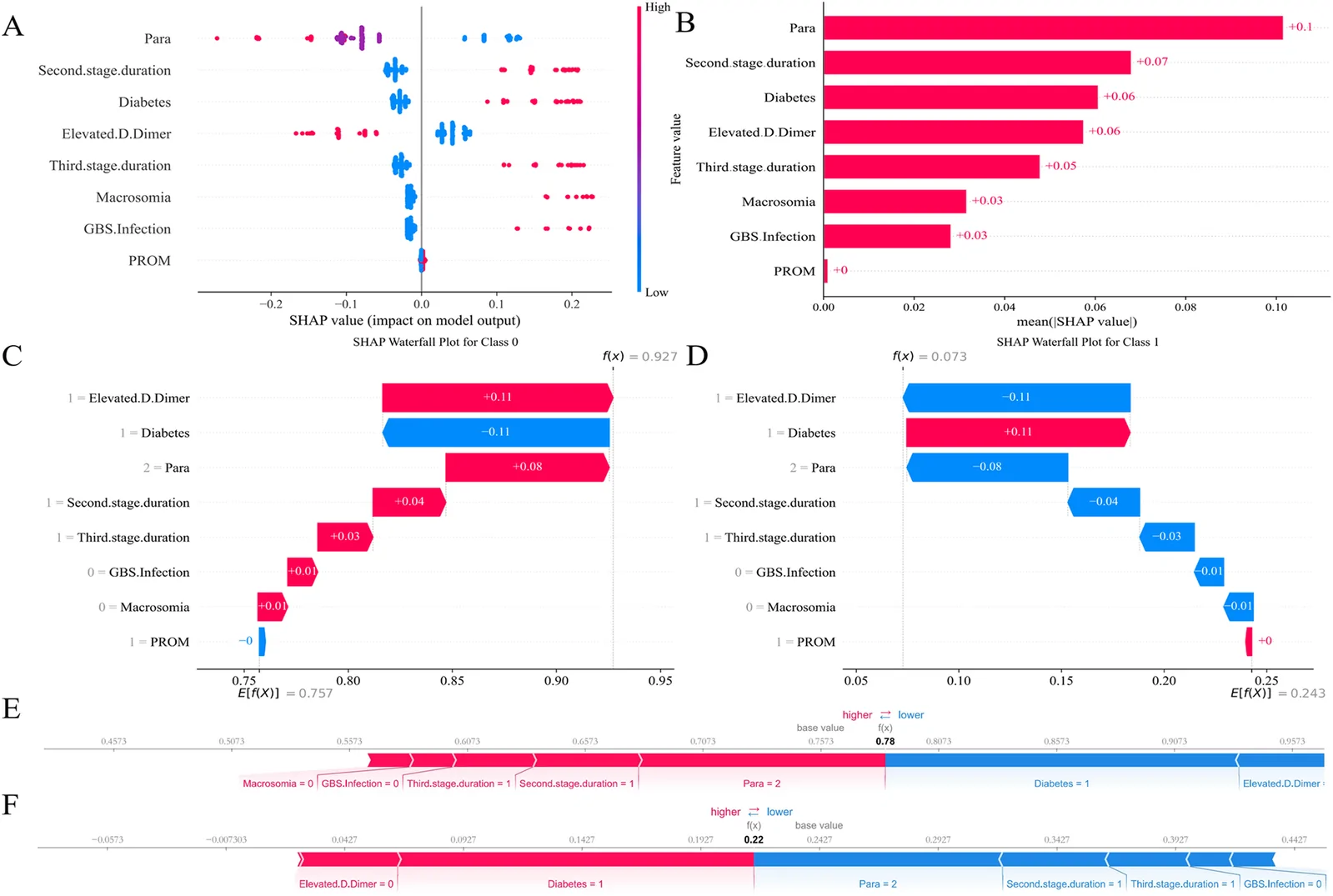
SHAP explainability analysis. **(A)** The beeswarm plot shows the SHAP values of each predictor and their association with PPH. **(B)** The mean absolute SHAP value of each feature reflects the degree of feature importance. **(C,D)** The waterfall plots intuitively show the cumulative contribution of each variable from baseline prediction to the final output in individual samples. **(E,F)** The force plots illustrate the contribution of each feature in individual samples, as well as its direction and magnitude of effect.

As shown in Figure 4, the SHAP algorithm was used to perform global interpretability analysis of the SVM prediction model, and a SHAP contribution plot was generated. The horizontal axis represents the SHAP value, which quantifies the impact of each feature on the model prediction. A larger SHAP value indicates a greater positive contribution of that feature to the prediction outcome. Single-sample SHAP explanations further provided waterfall and force plots for the SVM prediction model of PPH in advanced-maternal-age women, visually displaying the SHAP value of each feature and how the final prediction was determined. Blue and red bars represent protective factors and risk factors, respectively; longer bars indicate greater influence.

## 4 Discussion

This study systematically constructed and compared the predictive performance of seven machine learning models—LR, DT, RF, XGBoost, LightGBM, SVM, and ANN. Among these, the SVM model was selected as the clinically preferred model due to its highest sensitivity and F1-score. Compared with previously reported risk prediction models for PPH, our study achieved substantial improvements in three aspects: expansion of predictor dimensions, optimization of algorithmic performance, and enhancement of model interpretability (13, 14). Traditional PPH prediction models have mainly relied on prenatal static factors such as maternal age, parity, history of cesarean section, and placental abnormalities, which inherently belong to a static prediction framework. Such models fail to incorporate dynamic information during labor progression, including key variables such as gestational diabetes mellitus, duration of labor, and infection markers (15). In this study, we innovatively integrated intrapartum dynamic variables and laboratory indicators—including duration of the second and third stages of labor, Group B Streptococcus (GBS) infection status, and D-dimer levels—into the prediction system. This effectively expanded the temporal and informational dimensions of risk assessment and significantly improved the model's ability to identify PPH risk and provide early warning (16).

Although the AUC of SVM was not statistically different from other models, it achieved the highest sensitivity and F1-score. Given the severe consequences of missed PPH cases, we prioritized sensitivity and selected SVM as the clinically preferred model.

Furthermore, we applied the SHAP algorithm to provide global and individual-level visual explanations of the SVM model's predictions. This systematically quantified the marginal contribution of each predictor to the model output, effectively overcoming the methodological limitation of the “black box” nature inherent in traditional machine learning models, and provides transparent, intuitive, and quantifiable evidence to support clinical decision-making (17–19).

The SHAP analysis provided clinically actionable insights. Third stage of labor duration emerged as the strongest contributor to PPH risk. This finding reinforces that for women with a third stage exceeding 30 min, active management—including immediate oxytocin administration and controlled cord traction—should be initiated without waiting for visible hemorrhage. Elevated D-dimer, reflecting hyperfibrinolysis, highlights the need for early coagulation screening and consideration of prophylactic tranexamic acid in high-risk patients. GBS infection, as a potentially modifiable factor, underscores the potential benefit of intrapartum antibiotic prophylaxis beyond its established role in neonatal protection. Prolonged second stage and macrosomia reinforce the importance of preparedness for operative vaginal delivery and adequate staffing during high-risk labors. These SHAP-driven insights can guide bedside decision-making and facilitate shared decision-making with patients.

In the systematic comparison of seven machine learning models, the SVM model performed best on the validation set. This result can be explained from an algorithmic perspective. SVM uses the kernel trick to map the original low-dimensional feature space into a high-dimensional or even infinite-dimensional feature space, and then finds the optimal hyperplane with the maximum margin in that high-dimensional space, thereby accurately capturing complex decision boundaries. The algorithm is well-suited for high-dimensional, small-sample data. Through regularization parameters, it effectively controls model complexity, striking a balance between fitting the training data and maintaining generalization ability, thereby mitigating the risk of overfitting and ensuring robust extrapolation performance on an independent validation set. Compared with previous machine learning studies on PPH prediction, our SVM model showed comparable performance in terms of sensitivity and specificity, supporting its potential utility as a risk stratification tool (20).

The pathogenesis of PPH involves multi-system, multi-level complex pathophysiological mechanisms. Our study identified parity as an independent risk factor for PPH (21). The potential mechanisms may involve degenerative changes in uterine myofibers due to multiple pregnancies and deliveries, increased collagen deposition, progressive decline in uterine contractile reserve function, as well as uterine scar formation resulting from previous intrauterine procedures or abnormal placental attachment. GBS infection, as an important marker of intrauterine infection, exerts its pathogenic effects through multiple pathways: on one hand, GBS-induced local and systemic inflammatory responses release large amounts of pro-inflammatory cytokines (e.g., IL-6, TNF-α), which directly impair the contractile function of uterine smooth muscle cells and interfere with oxytocin receptor signaling pathways; on the other hand, infection-related vascular endothelial injury increases vascular permeability and disrupts the coagulation-fibrinolysis balance, thereby significantly increasing PPH risk (22, 23). GDM, as a potential risk factor for PPH, may involve several interrelated mechanisms: from the perspective of metabolic dysregulation, insulin resistance and the accompanying chronic low-grade inflammatory state can damage endothelial function and disturb the delicate balance between coagulation factor synthesis and fibrinolytic system activity; from the perspective of uterine contractile function, persistent hyperglycemia may reduce the sensitivity of uterine smooth muscle cells to oxytocin through pathways such as accumulation of advanced glycation end products (AGEs), leading to uterine atony and delayed hemostasis at the placental separation site (24, 25). D-dimer, a specific marker of fibrinolytic system activation, indicates a state of secondary hyperfibrinolysis. Elevated D-dimer levels suggest excessive consumption of coagulation factors and platelets, which impairs the physiological closure mechanism of the uterine placental bed sinusoids after delivery, resulting in compromised hemostatic function and significantly increasing the amount of blood loss and the risk of severe hemorrhagic events. Large-sample clinical studies by foreign investigators have further confirmed the positive correlation between D-dimer levels and the severity of PPH (26). Based on the above pathophysiological mechanisms, for high-risk pregnant women with GBS infection, GDM, or elevated D-dimer levels, intensified peripartum management should be implemented in clinical practice, including dynamic monitoring of relevant indicators before and during labor, advance preparation of individualized hemostatic strategies, and prophylactic use of uterine contracting agents such as oxytocin when necessary, to minimize the risk of PPH (27–30).

Abnormal duration of labor is also an important clinical factor contributing to PPH. Prolonged second stage of labor means that the fetal head exerts sustained pressure on the birth canal, leading to mechanical edema, ischemia, and even necrosis of the perineum, vagina, and cervix (31). After delivery, local vascular fragility increases, predisposing to hematoma formation and laceration bleeding. Meanwhile, prolonged and intense uterine contractions can deplete adenosine triphosphate (ATP), causing energy metabolism disorder and calcium homeostasis imbalance in uterine smooth muscle cells, which in turn leads to secondary uterine atony and further weakens the compression hemostasis effect of the uterus on the placental separation surface. Prolonged third stage of labor directly indicates pathological conditions such as delayed placental separation, retained placenta, or placental remnants, and is a strong warning sign for PPH (32, 33). It is also closely associated with an increased risk of long-term complications such as puerperal infection and late postpartum hemorrhage. Therefore, for women with signs of prolonged labor, clinicians should prepare uterotonics (e.g., oxytocin, carboprost), blood products (e.g., packed red blood cells, fresh frozen plasma) in advance, and actively assess the completeness of placental separation and uterine contraction status, so that timely targeted interventions can be undertaken (34).

Macrosomia is also an important risk factor for PPH. The underlying mechanisms mainly involve two aspects: first, macrosomia causes overdistension of the uterine cavity, leading to excessive stretching of uterine smooth muscle fibers, reduced postpartum myofiber retraction capacity, diminished contractile force, and delayed closure of the uterine placental bed sinuses; second, during vaginal delivery of a macrosomic infant, the mechanical tension generated as the fetal head and shoulders pass through the birth canal increases significantly, which can easily cause severe soft birth canal lacerations, including cervical lacerations, vaginal wall lacerations, and third- or fourth-degree perineal tears, directly disrupting local vascular integrity and inducing acute massive hemorrhage (35, 36). The specific molecular and pathophysiological mechanisms underlying these labor abnormalities and fetal factors—such as alterations in ion channel function in uterine smooth muscle cells and abnormal extracellular matrix remodeling—require further clarification through *in vivo* and *in vitro* experimental studies and multi-omics analyses (37).

From the perspective of clinical translation and application, the SVM model developed in this study demonstrates good predictive performance, suggesting that its potential clinical value lies in serving as an efficient screening tool to accurately identify parturients at low risk of postpartum hemorrhage (38–40). By concentrating limited healthcare resources on medium- to high-risk populations, this approach enables optimal allocation of medical resources, avoids unnecessary over-monitoring and intervention for low-risk individuals, and thereby improves the efficiency and cost-effectiveness of obstetric care while ensuring maternal and neonatal safety (41). However, it is important to recognize that PPH is a complex clinical event with significant heterogeneity, resulting from the interaction of multiple risk factors—maternal factors, fetal factors, labor factors, and medical interventions—which together introduce uncertainty and unpredictability (42–44). Relying solely on static indicators obtained at admission or prenatally for risk prediction cannot capture dynamic changes or emergent events during labor, and therefore has inherent limitations in predictive performance (45).

This study has several limitations that should be addressed in future research. First, the SVM model's sensitivity indicates that nearly half of PPH cases would be missed if used as a standalone screening tool. Therefore, we emphasize that this model should be viewed as an adjunctive risk-stratification aid rather than a replacement for clinical judgment. Its primary value lies in identifying a subset of women with relatively higher risk who may benefit from intensified surveillance, while low-risk predictions should still be interpreted with caution. Future work should incorporate real-time intrapartum monitoring data to improve sensitivity (46). Second, although the sample size is relatively large, the single-center retrospective design may introduce selection bias, and the external generalizability of the model needs to be further validated through multicenter, large-sample prospective studies (47). Third, while we used 5-fold cross-validation and an independent temporal validation set for dual evaluation of model performance, we did not use the bootstrap resampling method for internal validation, which may have some impact on the stability of performance estimates and the precision of confidence intervals (48, 49). Fourth, due to the limitations of the retrospective study design, we were unable to include intrapartum real-time dynamic monitoring indicators (e.g., uterine contraction pressure curves, Doppler ultrasound flow parameters) or novel biomarkers (e.g., microRNAs, metabolomic markers), leaving room for further expansion of the informational dimensions of predictors (50). Fifth, although our final model includes eight predictors, which is relatively parsimonious, it may still be valuable to develop an even simpler scoring tool or nomogram to further enhance practicality in rapid clinical decision-making scenarios. Sixth, although we assessed model calibration and clinical net benefit, we did not perform subgroup analyses (e.g., primiparous vs. multiparous women, vaginal delivery vs. cesarean section) nor conduct sensitivity analyses for missing proportions of key variables or outlier handling strategies (51). Finally, the LASSO-selected features can be clinically rationalized. Intrapartum dynamic variables (labor durations, GBS status) were selected because they directly reflect pathophysiological processes that influence uterine contractility and hemostasis—prolonged labor depletes ATP in uterine smooth muscle cells, impairing contractile function; GBS infection triggers inflammatory responses that interfere with oxytocin signaling. Static demographic factors (e.g., maternal age) were not selected because, in our AMA cohort with a narrow age range (35–45 years), these factors had limited discriminative power. Hypertension and mode of delivery were not selected because their effects may be mediated through pathways already captured by selected variables, or because of limited variability (all patients underwent vaginal delivery). Importantly, all selected variables are readily available during labor, making them practically useful for real-time risk assessment without requiring additional tests. The robustness of the model under different clinical scenarios requires further investigation. Subsequent studies will address these shortcomings through strategies such as multicenter external validation cohorts, incorporation of multi-dimensional dynamic monitoring data, and prospective cohort studies, with the aim of continuously optimizing model performance and advancing its translation into clinical decision support tools.

## 5 Conclusion

In this study, multiple machine learning algorithms were employed to develop predictive models for PPH risk in the population of women of AMA. Through systematic evaluation and comparison, the SVM model was selected as the clinically preferred model due to its highest sensitivity and F1-score. Key risk factors identified in this population include parity, prolonged duration of the second and third stages of labor, premature rupture of membranes, Group B Streptococcus infection, macrosomia, elevated D-dimer level, and gestational diabetes mellitus. After external validation in independent multicenter cohorts, clinicians may consider using this model as a supplementary screening tool, but it should not replace comprehensive clinical assessment. Pending such validation, the model's clinical deployment should be considered preliminary, and its use should be accompanied by local performance monitoring.

## Data Availability

The original contributions presented in the study are included in the article/Supplementary Material, further inquiries can be directed to the corresponding author.
