# Supplementary material for: Development and validation of a postpartum hemorrhage risk prediction model for advanced maternal age women undergoing vaginal delivery: a comparison of multiple machine learning algorithms
Source: Front Med (Lausanne). 2026 Sep 4;13:1898119. doi: 10.3389/fmed.2026.1898119 (PMC13585755; doi:10.3389/fmed.2026.1898119)
Supplement: Supplementary file 1 [file Datasheet1.docx]

import warnings

warnings.filterwarnings("ignore")

import os

import pandas as pd

from sklearn.preprocessing import StandardScaler

import statsmodels.api as sm

from sklearn.metrics import confusion_matrix, roc_curve, auc, roc_auc_score

import pickle

from sklearn.tree import DecisionTreeClassifier, plot_tree

from sklearn.ensemble import RandomForestClassifier

import numpy as np

from xgboost import XGBClassifier

import lightgbm as lgb

from sklearn.svm import SVC

from sklearn.neural_network import MLPClassifier

import matplotlib.pyplot as plt

from sklearn.metrics import brier_score_loss

from sklearn.utils import resample

import seaborn as sns

from scipy.stats import norm

from sklearn.calibration import calibration_curve

import shap

from statsmodels.nonparametric.smoothers_lowess import lowess

from sklearn.model_selection import GridSearchCV

from sklearn.metrics import cohen_kappa_score

os.getcwd()

plt.rcParams['font.family'] = 'Times New Roman'

train_data = pd.read_csv("traindata.csv", encoding="GBK")

val_data = pd.read_csv("valdata.csv", encoding="GBK")

from imblearn.over_sampling import SMOTE

X_origin = train_data.iloc[:, 1:]

y_origin = train_data.iloc[:, 0]

smote = SMOTE(sampling_strategy=0.8, random_state=123)

X_resampled, y_resampled = smote.fit_resample(X_origin, y_origin)

train_data_resampled = pd.concat([pd.Series(y_resampled, name=y_origin.name),

pd.DataFrame(X_resampled, columns=X_origin.columns)], axis=1)

train_data_resampled.to_csv("traindata_smote.csv", index=False)

print("Original class distribution:\n", y_origin.value_counts())

print("Class distribution after SMOTE:\n", y_resampled.value_counts())

train_data = pd.read_csv("traindata.csv", encoding="GBK")

print(train_data.info())

categorical_vars = train_data.iloc[:, 0:12].columns.tolist()

for var in categorical_vars:

train_data[var] = train_data[var].astype('category')

print(train_data.info())

X_train = train_data[['aCCI.score', 'SAS.score', 'Preoperative.K10',

'Preoperative.GO.SSS', 'Preoperative.OFI.8']]

X_train_const = sm.add_constant(X_train)

Y_train = train_data['Outcome']

logist_model = sm.Logit(Y_train, X_train_const).fit(disp=0)

logist_model.summary()

Y_train_logist = logist_model.predict(X_train_const)

auc_logist = roc_auc_score(Y_train, Y_train_logist)

auc_logist

with open("2.training_models/logistic_model.pkl", 'wb') as f:

pickle.dump(logist_model, f)

train_data_ml = pd.read_csv("traindata.csv", encoding="GBK")

val_data_ml = pd.read_csv("valdata.csv", encoding="GBK")

X_train = train_data[['aCCI.score', 'SAS.score', 'Preoperative.K10',

'Preoperative.GO.SSS', 'Preoperative.OFI.8']]

y_train = train_data_ml['Outcome']

X_val = val_data_ml[['aCCI.score', 'SAS.score', 'Preoperative.K10',

'Preoperative.GO.SSS', 'Preoperative.OFI.8']]

y_val = val_data_ml['Outcome']

os.environ['JOBLIB_TEMP_FOLDER'] = '/tmp'

tree_default = DecisionTreeClassifier(random_state=123)

tree_default.fit(X_train, y_train)

print("Decision tree default parameters:", pd.DataFrame.from_dict(tree_default.get_params(), orient='index'))

y_val_pred_prob_treed = tree_default.predict_proba(X_val)[:, 1]

auc_treed = roc_auc_score(y_val, y_val_pred_prob_treed)

print("Validation AUC of default decision tree:", auc_treed)

param_grid = {

'max_depth': [3, 5, 10, None],

'min_samples_split': [5, 10, 20],

'max_features': ['sqrt', None],

'ccp_alpha': [0.0, 0.01, 0.1]

}

grid_search_tree = GridSearchCV(

estimator=tree_default,

param_grid=param_grid,

scoring='roc_auc',

cv=5,

n_jobs=1,

verbose=1

)

grid_search_tree.fit(X_train, y_train)

best_auc_tree = grid_search_tree.best_score_

tree_model_best = grid_search_tree.best_estimator_

best_params = grid_search_tree.best_params_

print("Best decision tree parameters:", best_params)

print("Tuned decision tree detailed parameters:", pd.DataFrame.from_dict(tree_model_best.get_params(), orient='index'))

print("Cross-validation AUC of best tuned decision tree:", best_auc_tree)

print("Validation AUC of default decision tree:", auc_treed)

with open("2.training_models/tree_model.pkl", 'wb') as f:

pickle.dump(tree_model_best, f)

rf_model_default = RandomForestClassifier(random_state=123, oob_score=True)

rf_model_default.fit(X_train, y_train)

print("Random forest default parameters:", pd.DataFrame.from_dict(rf_model_default.get_params(), orient='index'))

y_val_pred_prob_rfd = rf_model_default.predict_proba(X_val)[:, 1]

auc_rfd = roc_auc_score(y_val, y_val_pred_prob_rfd)

print("Validation AUC of default random forest:", auc_rfd)

param_grid = {

'n_estimators': np.arange(50, 500, 50),

'max_features': list(range(2, round(np.sqrt(X_train.shape[1])) + 1))

}

grid_search_rf = GridSearchCV(

estimator=rf_model_default,

param_grid=param_grid,

scoring='roc_auc',

cv=5,

n_jobs=-1,

verbose=1

)

grid_search_rf.fit(X_train, y_train)

best_auc_rf = grid_search_rf.best_score_

rf_model_best = grid_search_rf.best_estimator_

best_params_rf = grid_search_rf.best_params_

best_ntree = best_params_rf['n_estimators']

best_mtry = best_params_rf['max_features']

print("Best random forest parameters: n_estimators =", best_ntree, ", max_features =", best_mtry)

print("Tuned random forest detailed parameters:", pd.DataFrame.from_dict(rf_model_best.get_params(), orient='index'))

print("Validation AUC of default random forest:", auc_rfd)

print("Cross-validation AUC of best tuned random forest:", best_auc_rf)

with open("2.training_models/rf_model.pkl", 'wb') as f:

pickle.dump(rf_model_best, f)

train_data_ml = pd.read_csv("traindata.csv", encoding="GBK")

val_data_ml = pd.read_csv("valdata.csv", encoding="GBK")

feature_cols = ['Gender', 'Drinking', 'Chronic.diseases', 'Dyslipidemia',

'SBP', 'Age', 'DBP', 'Cr', 'HDL.C', 'LDL.C',

'Hb', 'WC', 'FPG', 'TyG.index', 'TyG.BMI']

X_train = train_data_ml[feature_cols]

y_train = train_data_ml['Outcome']

X_val = val_data_ml[feature_cols]

y_val = val_data_ml['Outcome']

xgb_default = XGBClassifier(random_state=123, eval_metric='logloss')

xgb_default.fit(X_train, y_train)

y_val_pred_prob_xgbd = xgb_default.predict_proba(X_val)[:, 1]

auc_xgbd = roc_auc_score(y_val, y_val_pred_prob_xgbd)

print("Validation AUC of default XGBoost:", auc_xgbd)

param_grid = {

'learning_rate': [0.01, 0.1, 0.2],

'max_depth': [3, 5, 10],

'n_estimators': [50, 100, 200],

'subsample': [0.6, 0.8, 1.0]

}

grid_search_xgb = GridSearchCV(

estimator=xgb_default,

param_grid=param_grid,

scoring='roc_auc',

cv=5,

n_jobs=-1,

verbose=1

)

grid_search_xgb.fit(X_train, y_train)

best_auc_xgb = grid_search_xgb.best_score_

xgb_model_best = grid_search_xgb.best_estimator_

best_params_xgb = grid_search_xgb.best_params_

print("Best XGBoost parameters:", best_params_xgb)

print("Tuned XGBoost detailed parameters:", pd.DataFrame.from_dict(xgb_model_best.get_params(), orient='index'))

print("Validation AUC of default XGBoost:", auc_xgbd)

print("Cross-validation AUC of best tuned XGBoost:", best_auc_xgb)

with open("2.training_models/xgb_model.pkl", 'wb') as f:

pickle.dump(xgb_model_best, f)

lgb_default = lgb.LGBMClassifier(random_state=123)

lgb_default.fit(X_train, y_train)

y_val_pred_prob_lgbd = lgb_default.predict_proba(X_val)[:, 1]

auc_lgbd = roc_auc_score(y_val, y_val_pred_prob_lgbd)

print("Validation AUC of default LightGBM:", auc_lgbd)

param_grid = {

'learning_rate': [0.1, 0.2],

'num_leaves': [31, 50, 100],

'n_estimators': [100],

'subsample': [0.6, 0.8, 1.0],

'colsample_bytree': [0.6, 0.8, 1.0]

}

grid_search_lgb = GridSearchCV(

estimator=lgb_default,

param_grid=param_grid,

scoring='roc_auc',

cv=5,

n_jobs=-1,

verbose=1

)

grid_search_lgb.fit(X_train, y_train)

best_auc_lgb = grid_search_lgb.best_score_

lgb_model_best = grid_search_lgb.best_estimator_

best_params_lgb = grid_search_lgb.best_params_

print("Best LightGBM parameters:", best_params_lgb)

print("Tuned LightGBM detailed parameters:", pd.DataFrame.from_dict(lgb_model_best.get_params(), orient='index'))

print("Validation AUC of default LightGBM:", auc_lgbd)

print("Cross-validation AUC of best tuned LightGBM:", best_auc_lgb)

with open("2.training_models/lgb_model.pkl", 'wb') as f:

pickle.dump(lgb_model_best, f)

svm_default = SVC(probability=True, random_state=123)

svm_default.fit(X_train, y_train)

y_val_pred_prob_svmd = svm_default.predict_proba(X_val)[:, 1]

auc_svmd = roc_auc_score(y_val, y_val_pred_prob_svmd)

print("Validation AUC of default SVM:", auc_svmd)

param_grid = {

'C': [0.1],

'kernel': ['linear', 'rbf'],

'gamma': ['scale', 'auto', 0.01],

'degree': [2, 3, 4]

}

grid_search_svm = GridSearchCV(

estimator=svm_default,

param_grid=param_grid,

scoring='roc_auc',

cv=5,

n_jobs=-1,

verbose=1

)

grid_search_svm.fit(X_train, y_train)

best_auc_svm = grid_search_svm.best_score_

svm_model_best = grid_search_svm.best_estimator_

best_params_svm = grid_search_svm.best_params_

print("Best SVM parameters:", best_params_svm)

print("Tuned SVM detailed parameters:", pd.DataFrame.from_dict(svm_model_best.get_params(), orient='index'))

print("Validation AUC of default SVM:", auc_svmd)

print("Cross-validation AUC of best tuned SVM:", best_auc_svm)

with open("2.training_models/svm_model.pkl", 'wb') as f:

pickle.dump(svm_model_best, f)

ann_default = MLPClassifier(random_state=123, max_iter=500)

ann_default.fit(X_train, y_train)

y_val_pred_prob_annd = ann_default.predict_proba(X_val)[:, 1]

auc_annd = roc_auc_score(y_val, y_val_pred_prob_annd)

print("Validation AUC of default ANN:", auc_annd)

param_grid = {

'hidden_layer_sizes': [(25,), (50,), (100,), (10, 10), (50, 50), (100, 100), (50, 50, 50)],

'activation': ['relu', 'tanh', 'logistic']

}

grid_search_ann = GridSearchCV(

estimator=ann_default,

param_grid=param_grid,

scoring='roc_auc',

cv=5,

n_jobs=-1,

verbose=1

)

grid_search_ann.fit(X_train, y_train)

best_auc_ann = grid_search_ann.best_score_

ann_model_best = grid_search_ann.best_estimator_

best_params_ann = grid_search_ann.best_params_

print("Best ANN parameters:", best_params_ann)

print("Tuned ANN detailed parameters:", pd.DataFrame.from_dict(ann_model_best.get_params(), orient='index'))

print("Validation AUC of default ANN:", auc_annd)

print("Cross-validation AUC of best tuned ANN:", best_auc_ann)

with open("2.training_models/ann_model.pkl", 'wb') as f:

pickle.dump(ann_model_best, f)

val_data = pd.read_csv("valdata.csv", encoding="GBK")

X_val_logist = val_data[['Gender', 'Drinking', 'Chronic.diseases', 'Dyslipidemia',

'SBP', 'Age', 'DBP', 'Cr', 'HDL.C', 'LDL.C',

'Hb', 'WC', 'FPG', 'TyG.index', 'TyG.BMI']]

X_val_logist_const = sm.add_constant(X_val_logist)

y_val_logist = val_data['Outcome']

categorical_vars = val_data.iloc[:, 0:7].columns.tolist()

for var in categorical_vars:

val_data[var] = val_data[var].astype('category')

print(val_data.info())

val_data_ml = pd.read_csv("valdata.csv", encoding="GBK")

X_val = val_data_ml[['Gender', 'Drinking', 'Chronic.diseases', 'Dyslipidemia',

'SBP', 'Age', 'DBP', 'Cr', 'HDL.C', 'LDL.C',

'Hb', 'WC', 'FPG', 'TyG.index', 'TyG.BMI']]

y_val = val_data_ml['Outcome']

with open("2.training_models/logistic_model.pkl", 'rb') as f:

logist_model = pickle.load(f)

with open("2.training_models/tree_model.pkl", 'rb') as f:

tree_model = pickle.load(f)

with open("2.training_models/rf_model.pkl", 'rb') as f:

rf_model = pickle.load(f)

with open("2.training_models/xgb_model.pkl", 'rb') as f:

xgb_model = pickle.load(f)

with open("2.training_models/lgb_model.pkl", 'rb') as f:

lgb_model = pickle.load(f)

with open("2.training_models/svm_model.pkl", 'rb') as f:

svm_model = pickle.load(f)

with open("2.training_models/ann_model.pkl", 'rb') as f:

ann_model = pickle.load(f)

y_val_pred_prob_logist = logist_model.predict(X_val_logist_const)

y_val_pred_logist = (y_val_pred_prob_logist >= 0.5).astype(int)

y_val_pred_prob_tree = tree_model.predict_proba(X_val)[:, 1]

y_val_pred_tree = (y_val_pred_prob_tree >= 0.5).astype(int)

y_val_pred_prob_rf = rf_model.predict_proba(X_val)[:, 1]

y_val_pred_rf = (y_val_pred_prob_rf >= 0.5).astype(int)

y_val_pred_prob_xgb = xgb_model.predict_proba(X_val)[:, 1]

y_val_pred_xgb = (y_val_pred_prob_xgb >= 0.5).astype(int)

y_val_pred_prob_lgb = lgb_model.predict_proba(X_val)[:, 1]

y_val_pred_lgb = (y_val_pred_prob_lgb >= 0.5).astype(int)

y_val_pred_prob_svm = svm_model.predict_proba(X_val)[:, 1]

y_val_pred_svm = (y_val_pred_prob_svm >= 0.5).astype(int)

y_val_pred_prob_ann = ann_model.predict_proba(X_val)[:, 1]

y_val_pred_ann = (y_val_pred_prob_ann >= 0.5).astype(int)

output_folder = "3.validation_confusion_matrix"

def CM_plot(cm, model_name):

plt.figure(figsize=(5, 4))

cm_percentage = cm.astype('float') / cm.sum(axis=1)[:, np.newaxis]

annotations = np.empty_like(cm).astype(str)

for i in range(cm.shape[0]):

for j in range(cm.shape[1]):

annotations[i, j] = f"{cm[i, j]}\n{cm_percentage[i, j]:.2%}"

sns.heatmap(cm_percentage, annot=annotations, fmt="", cmap="Blues", xticklabels=['No Outcome', 'Outcome'], yticklabels=['No Outcome', 'Outcome'])

plt.xlabel("Predicted")

plt.ylabel("Actual")

plt.title(f"Confusion Matrix - {model_name}")

plt.savefig(os.path.join(output_folder, f"{model_name}_confusion_matrix.pdf"), bbox_inches='tight')

plt.show()

cm_logist = confusion_matrix(y_val_logist, y_val_pred_logist)

CM_plot(cm_logist, "Logistic")

cm_tree = confusion_matrix(y_val, y_val_pred_tree)

CM_plot(cm_tree, "DecisionTree")

cm_rf = confusion_matrix(y_val, y_val_pred_rf)

CM_plot(cm_rf, "RandomForest")

cm_xgb = confusion_matrix(y_val, y_val_pred_xgb)

CM_plot(cm_xgb, "XGBoost")

cm_lgb = confusion_matrix(y_val, y_val_pred_lgb)

CM_plot(cm_lgb, "LightGBM")

cm_svm = confusion_matrix(y_val, y_val_pred_svm)

CM_plot(cm_svm, "SVM")

cm_ann = confusion_matrix(y_val, y_val_pred_ann)

CM_plot(cm_ann, "ANN")

def calculate_acc_pre_sen_f1_spc(cm, y_true, y_pred):

tn, fp, fn, tp = cm.ravel()

accuracy = (tp + tn) / (tp + fn + tn + fp)

precision = tp / (tp + fp)

sensitivity = tp / (tp + fn)

f1score	= 2 * (precision * sensitivity) / (precision + sensitivity)

specificity = tn / (tn + fp)

kappa = cohen_kappa_score(y_true, y_pred)

youden = sensitivity + specificity - 1

ppv = precision

npv = tn / (tn + fn)

return accuracy, precision, sensitivity, f1score, specificity, kappa, youden, ppv, npv

accuracy_logist, precision_logist, sensitivity_logist, f1_logist, specificity_logist, kappa_logist, youden_logist, ppv_logist, npv_logist = calculate_acc_pre_sen_f1_spc(cm_logist, y_val_logist, y_val_pred_logist)

print(f"Logistic Model -> Accuracy: {accuracy_logist:.3f}, Precision: {precision_logist:.3f}, Sensitivity: {sensitivity_logist:.3f}, F1 Score: {f1_logist:.3f}, Specificity: {specificity_logist:.3f}, Kappa: {kappa_logist:.3f}, Youden's J: {youden_logist:.3f}, PPV: {ppv_logist:.3f}, NPV: {npv_logist:.3f}")

accuracy_tree, precision_tree, sensitivity_tree, f1_tree, specificity_tree, kappa_tree, youden_tree, ppv_tree, npv_tree = calculate_acc_pre_sen_f1_spc(cm_tree, y_val, y_val_pred_tree)

print(f"Decision Tree Model -> Accuracy: {accuracy_tree:.3f}, Precision: {precision_tree:.3f}, Sensitivity: {sensitivity_tree:.3f}, F1 Score: {f1_tree:.3f}, Specificity: {specificity_tree:.3f}, Kappa: {kappa_tree:.3f}, Youden's J: {youden_tree:.3f}, PPV: {ppv_tree:.3f}, NPV: {npv_tree:.3f}")

accuracy_rf, precision_rf, sensitivity_rf, f1_rf, specificity_rf, kappa_rf, youden_rf, ppv_rf, npv_rf = calculate_acc_pre_sen_f1_spc(cm_rf, y_val, y_val_pred_rf)

print(f"Random Forest Model -> Accuracy: {accuracy_rf:.3f}, Precision: {precision_rf:.3f}, Sensitivity: {sensitivity_rf:.3f}, F1 Score: {f1_rf:.3f}, Specificity: {specificity_rf:.3f}, Kappa: {kappa_rf:.3f}, Youden's J: {youden_rf:.3f}, PPV: {ppv_rf:.3f}, NPV: {npv_rf:.3f}")

accuracy_xgb, precision_xgb, sensitivity_xgb, f1_xgb, specificity_xgb, kappa_xgb, youden_xgb, ppv_xgb, npv_xgb = calculate_acc_pre_sen_f1_spc(cm_xgb, y_val, y_val_pred_xgb)

print(f"XGBoost Model -> Accuracy: {accuracy_xgb:.3f}, Precision: {precision_xgb:.3f}, Sensitivity: {sensitivity_xgb:.3f}, F1 Score: {f1_xgb:.3f}, Specificity: {specificity_xgb:.3f}, Kappa: {kappa_xgb:.3f}, Youden's J: {youden_xgb:.3f}, PPV: {ppv_xgb:.3f}, NPV: {npv_xgb:.3f}")

accuracy_lgb, precision_lgb, sensitivity_lgb, f1_lgb, specificity_lgb, kappa_lgb, youden_lgb, ppv_lgb, npv_lgb = calculate_acc_pre_sen_f1_spc(cm_lgb, y_val, y_val_pred_lgb)

print(f"LightGBM Model -> Accuracy: {accuracy_lgb:.3f}, Precision: {precision_lgb:.3f}, Sensitivity: {sensitivity_lgb:.3f}, F1 Score: {f1_lgb:.3f}, Specificity: {specificity_lgb:.3f}, Kappa: {kappa_lgb:.3f}, Youden's J: {youden_lgb:.3f}, PPV: {ppv_lgb:.3f}, NPV: {npv_lgb:.3f}")

accuracy_svm, precision_svm, sensitivity_svm, f1_svm, specificity_svm, kappa_svm, youden_svm, ppv_svm, npv_svm = calculate_acc_pre_sen_f1_spc(cm_svm, y_val, y_val_pred_svm)

print(f"SVM Model -> Accuracy: {accuracy_svm:.3f}, Precision: {precision_svm:.3f}, Sensitivity: {sensitivity_svm:.3f}, F1 Score: {f1_svm:.3f}, Specificity: {specificity_svm:.3f}, Kappa: {kappa_svm:.3f}, Youden's J: {youden_svm:.3f}, PPV: {ppv_svm:.3f}, NPV: {npv_svm:.3f}")

accuracy_ann, precision_ann, sensitivity_ann, f1_ann, specificity_ann, kappa_ann, youden_ann, ppv_ann, npv_ann = calculate_acc_pre_sen_f1_spc(cm_ann, y_val, y_val_pred_ann)

print(f"ANN Model -> Accuracy: {accuracy_ann:.3f}, Precision: {precision_ann:.3f}, Sensitivity: {sensitivity_ann:.3f}, F1 Score: {f1_ann:.3f}, Specificity: {specificity_ann:.3f}, Kappa: {kappa_ann:.3f}, Youden's J: {youden_ann:.3f}, PPV: {ppv_ann:.3f}, NPV: {npv_ann:.3f}")

def calculate_auc(y_label, y_pred_prob, n_boot=1000, random_state=123):

auc_value = roc_auc_score(y_label, y_pred_prob)

data = np.column_stack([y_label, y_pred_prob])

rng = np.random.default_rng(random_state)

auc_boots = []

for _ in range(n_boot):

idx = rng.choice(len(data), size=len(data), replace=True)

y_label_boot = data[idx, 0]

y_pred_boot = data[idx, 1]

if len(np.unique(y_label_boot)) < 2:

continue

auc_boot = roc_auc_score(y_label_boot, y_pred_boot)

auc_boots.append(auc_boot)

auc_ci_lower, auc_ci_upper = np.percentile(auc_boots, [2.5, 97.5])

return auc_value, auc_ci_lower, auc_ci_upper

auc_value_logist, auc_ci_lower_logist, auc_ci_upper_logist = calculate_auc(y_val_logist, y_val_pred_prob_logist)

print(f"Logistic Model AUC: {auc_value_logist:.3f} (95% CI: {auc_ci_lower_logist:.3f} - {auc_ci_upper_logist:.3f})")

auc_value_tree, auc_ci_lower_tree, auc_ci_upper_tree = calculate_auc(y_val, y_val_pred_prob_tree)

print(f"Decision Tree Model AUC: {auc_value_tree:.3f} (95% CI: {auc_ci_lower_tree:.3f} - {auc_ci_upper_tree:.3f})")

auc_value_rf, auc_ci_lower_rf, auc_ci_upper_rf = calculate_auc(y_val, y_val_pred_prob_rf)

print(f"Random Forest Model AUC: {auc_value_rf:.3f} (95% CI: {auc_ci_lower_rf:.3f} - {auc_ci_upper_rf:.3f})")

auc_value_xgb, auc_ci_lower_xgb, auc_ci_upper_xgb = calculate_auc(y_val, y_val_pred_prob_xgb)

print(f"XGBoost Model AUC: {auc_value_xgb:.3f} (95% CI: {auc_ci_lower_xgb:.3f} - {auc_ci_upper_xgb:.3f})")

auc_value_lgb, auc_ci_lower_lgb, auc_ci_upper_lgb = calculate_auc(y_val, y_val_pred_prob_lgb)

print(f"LightGBM Model AUC: {auc_value_lgb:.3f} (95% CI: {auc_ci_lower_lgb:.3f} - {auc_ci_upper_lgb:.3f})")

auc_value_svm, auc_ci_lower_svm, auc_ci_upper_svm = calculate_auc(y_val, y_val_pred_prob_svm)

print(f"SVM Model AUC: {auc_value_svm:.3f} (95% CI: {auc_ci_lower_svm:.3f} - {auc_ci_upper_svm:.3f})")

auc_value_ann, auc_ci_lower_ann, auc_ci_upper_ann = calculate_auc(y_val, y_val_pred_prob_ann)

print(f"ANN Model AUC: {auc_value_ann:.3f} (95% CI: {auc_ci_lower_ann:.3f} - {auc_ci_upper_ann:.3f})")

model_results_validation = pd.DataFrame({

"Model": ["Logistic", "Decision Tree", "Random Forest", "XGBoost", "LightGBM", "SVM", "ANN"],

"AUC": [auc_value_logist, auc_value_tree, auc_value_rf, auc_value_xgb, auc_value_lgb, auc_value_svm, auc_value_ann],

"95% CI Lower": [auc_ci_lower_logist, auc_ci_lower_tree, auc_ci_lower_rf, auc_ci_lower_xgb, auc_ci_lower_lgb, auc_ci_lower_svm, auc_ci_lower_ann],

"95% CI Upper": [auc_ci_upper_logist, auc_ci_upper_tree, auc_ci_upper_rf, auc_ci_upper_xgb, auc_ci_upper_lgb, auc_ci_upper_svm, auc_ci_upper_ann],

"Accuracy": [accuracy_logist, accuracy_tree, accuracy_rf, accuracy_xgb, accuracy_lgb, accuracy_svm, accuracy_ann],

"Precision": [precision_logist, precision_tree, precision_rf, precision_xgb, precision_lgb, precision_svm, precision_ann],

"Sensitivity": [sensitivity_logist, sensitivity_tree, sensitivity_rf, sensitivity_xgb, sensitivity_lgb, sensitivity_svm, sensitivity_ann],

"Specificity": [specificity_logist, specificity_tree, specificity_rf, specificity_xgb, specificity_lgb, specificity_svm, specificity_ann],

"F1 Score": [f1_logist, f1_tree, f1_rf, f1_xgb, f1_lgb, f1_svm, f1_ann],

"Kappa": [kappa_logist, kappa_tree, kappa_rf, kappa_xgb, kappa_lgb, kappa_svm, kappa_ann],

"Youden's J": [youden_logist, youden_tree, youden_rf, youden_xgb, youden_lgb, youden_svm, youden_ann],

"PPV": [ppv_logist, ppv_tree, ppv_rf, ppv_xgb, ppv_lgb, ppv_svm, ppv_ann],

"NPV": [npv_logist, npv_tree, npv_rf, npv_xgb, npv_lgb, npv_svm, npv_ann]

})

model_results_validation

model_results_validation.to_csv("4.validation_model_evaluation/model_performance_validation.csv", index=False)

output_folder = "4.validation_model_evaluation/single_model_evaluation"

def ROC_plot(y_label, y_pred_prob, auc_value, auc_ci_lower, auc_ci_upper, model_name):

fpr, tpr, _ = roc_curve(y_label, y_pred_prob)

plt.figure(figsize=(6, 5))

plt.plot(fpr, tpr, label=f'AUC = {auc_value:.3f} (95% CI: {auc_ci_lower:.3f} - {auc_ci_upper:.3f})')

plt.plot([0, 1], [0, 1], linestyle='--', color='gray')

plt.xlabel("1-Specificity")

plt.ylabel("Sensitivity")

plt.title("ROC Curve")

plt.legend(loc='lower right')

plt.savefig(os.path.join(output_folder, f"{model_name}_ROC_curve.pdf"), bbox_inches='tight')

plt.show()

ROC_plot(y_val_logist, y_val_pred_prob_logist, auc_value_logist, auc_ci_lower_logist, auc_ci_upper_logist, "Logistic")

ROC_plot(y_val, y_val_pred_prob_tree, auc_value_tree, auc_ci_lower_tree, auc_ci_upper_tree, "DecisionTree")

ROC_plot(y_val, y_val_pred_prob_rf, auc_value_rf, auc_ci_lower_rf, auc_ci_upper_rf, "RandomForest")

ROC_plot(y_val, y_val_pred_prob_xgb, auc_value_xgb, auc_ci_lower_xgb, auc_ci_upper_xgb, "XGBoost")

ROC_plot(y_val, y_val_pred_prob_lgb, auc_value_lgb, auc_ci_lower_lgb, auc_ci_upper_lgb, "LightGBM")

ROC_plot(y_val, y_val_pred_prob_svm, auc_value_svm, auc_ci_lower_svm, auc_ci_upper_svm, "SVM")

ROC_plot(y_val, y_val_pred_prob_ann, auc_value_ann, auc_ci_lower_ann, auc_ci_upper_ann, "ANN")

plt.figure(figsize=(8, 6))

models = {

"Logistic": (y_val_logist, y_val_pred_prob_logist, auc_value_logist, auc_ci_lower_logist, auc_ci_upper_logist),

"Decision Tree": (y_val, y_val_pred_prob_tree, auc_value_tree, auc_ci_lower_tree, auc_ci_upper_tree),

"Random Forest": (y_val, y_val_pred_prob_rf, auc_value_rf, auc_ci_lower_rf, auc_ci_upper_rf),

"XGBoost": (y_val, y_val_pred_prob_xgb, auc_value_xgb, auc_ci_lower_xgb, auc_ci_upper_xgb),

"LightGBM": (y_val, y_val_pred_prob_lgb, auc_value_lgb, auc_ci_lower_lgb, auc_ci_upper_lgb),

"SVM": (y_val, y_val_pred_prob_svm, auc_value_svm, auc_ci_lower_svm, auc_ci_upper_svm),

"ANN": (y_val, y_val_pred_prob_ann, auc_value_ann, auc_ci_lower_ann, auc_ci_upper_ann)

}

for model_name, (y_true, y_pred_prob, auc_value, ci_lower, ci_upper) in models.items():

fpr, tpr, _ = roc_curve(y_true, y_pred_prob)

plt.plot(fpr, tpr, label=f"{model_name} (AUC = {auc_value:.3f}, 95% CI: {ci_lower:.3f}-{ci_upper:.3f})")

plt.plot([0, 1], [0, 1], linestyle='--', color='gray')

plt.xlabel("1-Specificity")

plt.ylabel("Sensitivity")

plt.title("ROC Curve - Validation Set Model Comparison")

plt.legend(loc='lower right')

plt.grid()

plt.savefig("4.validation_model_evaluation/ROC_curves_allmodel_validation.pdf", dpi=300)

plt.show()

output_folder = "4.validation_model_evaluation/single_model_evaluation"

def brier_score_confidence_interval(y_true, y_pred_prob, n_bootstraps=1000, alpha=0.05):

brier_scores = []

for _ in range(n_bootstraps):

indices = resample(np.arange(len(y_true)), n_samples=len(y_true), replace=True)

y_true_sample = np.array(y_true)[indices]

y_pred_prob_sample = np.array(y_pred_prob)[indices]

brier_score = np.mean((y_pred_prob_sample - y_true_sample) ** 2)

brier_scores.append(brier_score)

brier_mean = np.mean(brier_scores)

brier_lower = np.percentile(brier_scores, (alpha / 2) * 100)

brier_upper = np.percentile(brier_scores, (1 - alpha / 2) * 100)

return brier_mean, brier_lower, brier_upper

def CaliC_plot(y_label, y_pred_prob, model_name, n_bins=10):

prob_true, prob_pred = calibration_curve(y_label, y_pred_prob, n_bins=n_bins)

brier_mean, brier_lower, brier_upper = brier_score_confidence_interval(y_label, y_pred_prob)

plt.figure(figsize=(6, 5))

plt.plot(prob_pred, prob_true, marker='o', label=f'Calibration curve (Brier Score: {brier_mean:.3f} [95% CI: {brier_lower:.3f} - {brier_upper:.3f}])')

plt.plot([0, 1], [0, 1], linestyle='--', color='gray', label='Perfect calibration')

plt.xlabel("Predicted Probability")

plt.ylabel("True Probability")

plt.title(f"Calibration Curve - {model_name}")

plt.legend(loc='lower right')

plt.savefig(os.path.join(output_folder, f"{model_name}_Calibration_Curve.pdf"), bbox_inches='tight')

plt.show()

CaliC_plot(y_val_logist, y_val_pred_prob_logist, "Logistic")

CaliC_plot(y_val, y_val_pred_prob_tree, "DecisionTree")

CaliC_plot(y_val, y_val_pred_prob_rf, "RandomForest")

CaliC_plot(y_val, y_val_pred_prob_xgb, "XGBoost")

CaliC_plot(y_val, y_val_pred_prob_lgb, "LightGBM")

CaliC_plot(y_val, y_val_pred_prob_svm, "SVM")

CaliC_plot(y_val, y_val_pred_prob_ann, "ANN")

plt.figure(figsize=(10, 8))

models = {

"Logistic": (y_val_logist, y_val_pred_prob_logist),

"Decision Tree": (y_val, y_val_pred_prob_tree),

"Random Forest": (y_val, y_val_pred_prob_rf),

"XGBoost": (y_val, y_val_pred_prob_xgb),

"LightGBM": (y_val, y_val_pred_prob_lgb),

"SVM": (y_val, y_val_pred_prob_svm),

"ANN": (y_val, y_val_pred_prob_ann)

}

for model_name, (y_true, y_pred_prob) in models.items():

prob_true, prob_pred = calibration_curve(y_true, y_pred_prob, n_bins=10)

brier_mean, brier_lower, brier_upper = brier_score_confidence_interval(y_true, y_pred_prob)

plt.plot(prob_pred, prob_true, marker='o', label=f'{model_name} (Brier Score: {brier_mean:.3f} [95% CI: {brier_lower:.3f} - {brier_upper:.3f}])')

plt.plot([0, 1], [0, 1], linestyle='--', color='gray', label='Perfect Calibration')

plt.xlabel("Predicted Probability")

plt.ylabel("True Probability")

plt.title("Calibration Curves - Validation Set Model Comparison")

plt.legend(loc='lower right')

plt.grid()

plt.savefig("4.validation_model_evaluation/Calibration_curves_allmodel_validation.pdf", dpi=300)

plt.show()

output_folder = "4.validation_model_evaluation/single_model_evaluation"

def calculate_net_benefit(y_label, y_pred_prob, thresholds=np.linspace(0.01, 1, 100)):

net_benefit_model = []

net_benefit_alltrt = []

net_benefits_notrt = [0] * len(thresholds)

total_obs = len(y_label)

for thresh in thresholds:

y_pred_label = y_pred_prob > thresh

tn, fp, fn, tp = confusion_matrix(y_label, y_pred_label).ravel()

net_benefit = (tp / total_obs) - (fp / total_obs) * (thresh / (1 - thresh))

net_benefit_model.append(net_benefit)

tn, fp, fn, tp = confusion_matrix(y_label, y_label).ravel()

total_right = tp + tn

net_benefit = (tp / total_right) - (tn / total_right) * (thresh / (1 - thresh))

net_benefit_alltrt.append(net_benefit)

return net_benefit_model, net_benefit_alltrt, net_benefits_notrt

def DCA_plot(net_benefit_model, net_benefit_alltrt, net_benefits_notrt, model_name, thresholds=np.linspace(0.01, 0.99, 100)):

plt.figure(figsize=(8, 6))

plt.plot(thresholds, net_benefit_model, label="Model Net Benefit", color='blue', linewidth=2)

plt.plot(thresholds, net_benefit_alltrt, label="Treat All", color="red", linewidth=2)

plt.plot(thresholds, net_benefits_notrt, linestyle='--', color='green', label="Treat None", linewidth=2)

plt.xlabel("Threshold Probability")

plt.ylim(-0.15, np.nanmax(np.array(net_benefit_model)) + 0.1)

plt.ylabel("Net Benefit")

plt.title(f"Decision Curve Analysis - {model_name}")

plt.legend()

plt.grid(True)

plt.savefig(os.path.join(output_folder, f"{model_name}_DCA_curve.pdf"), bbox_inches='tight')

plt.show()

net_benefit_logist, net_benefit_alltrt_logist, net_benefits_notrt_logist = calculate_net_benefit(y_val_logist, y_val_pred_prob_logist)

DCA_plot(net_benefit_logist, net_benefit_alltrt_logist, net_benefits_notrt_logist, "Logistic")

net_benefit_tree, net_benefit_alltrt_tree, net_benefits_notrt_tree = calculate_net_benefit(y_val, y_val_pred_prob_tree)

DCA_plot(net_benefit_tree, net_benefit_alltrt_tree, net_benefits_notrt_tree, "DecisionTree")

net_benefit_rf, net_benefit_alltrt_rf, net_benefits_notrt_rf = calculate_net_benefit(y_val, y_val_pred_prob_rf)

DCA_plot(net_benefit_rf, net_benefit_alltrt_rf, net_benefits_notrt_rf, "RandomForest")

net_benefit_xgb, net_benefit_alltrt_xgb, net_benefits_notrt_xgb = calculate_net_benefit(y_val, y_val_pred_prob_xgb)

DCA_plot(net_benefit_xgb, net_benefit_alltrt_xgb, net_benefits_notrt_xgb, "XGBoost")

net_benefit_lgb, net_benefit_alltrt_lgb, net_benefits_notrt_lgb = calculate_net_benefit(y_val, y_val_pred_prob_lgb)

DCA_plot(net_benefit_lgb, net_benefit_alltrt_lgb, net_benefits_notrt_lgb, "LightGBM")

net_benefit_svm, net_benefit_alltrt_svm, net_benefits_notrt_svm = calculate_net_benefit(y_val, y_val_pred_prob_svm)

DCA_plot(net_benefit_svm, net_benefit_alltrt_svm, net_benefits_notrt_svm, "SVM")

net_benefit_ann, net_benefit_alltrt_ann, net_benefits_notrt_ann = calculate_net_benefit(y_val, y_val_pred_prob_ann)

DCA_plot(net_benefit_ann, net_benefit_alltrt_ann, net_benefits_notrt_ann, "ANN")

plt.figure(figsize=(10, 8))

thresholds = np.linspace(0.01, 0.99, 100)

models = {

"Logistic": (y_val_logist, y_val_pred_prob_logist),

"Decision Tree": (y_val, y_val_pred_prob_tree),

"Random Forest": (y_val, y_val_pred_prob_rf),

"XGBoost": (y_val, y_val_pred_prob_xgb),

"LightGBM": (y_val, y_val_pred_prob_lgb),

"SVM": (y_val, y_val_pred_prob_svm),

"ANN": (y_val, y_val_pred_prob_ann)

}

all_net_benefits = {}

for model_name, (y_true, y_pred_prob) in models.items():

net_benefit, _, _ = calculate_net_benefit(y_true, y_pred_prob)

all_net_benefits[model_name] = net_benefit

for model_name, net_benefit in all_net_benefits.items():

plt.plot(thresholds, net_benefit, label=model_name)

plt.plot(thresholds, net_benefit_alltrt_logist, linestyle="--", color="red", label="Treat All")

plt.plot(thresholds, net_benefits_notrt_logist, linestyle="--", color="green", label="Treat None")

plt.xlabel("Threshold Probability")

plt.ylim(-0.3, np.nanmax(np.array([max(net_benefit) for net_benefit in all_net_benefits.values()])) + 0.1)

plt.ylabel("Net Benefit")

plt.title("Decision Curve Analysis - Validation Set Model Comparison")

plt.legend(loc="lower right", fontsize=12)

plt.grid()

plt.savefig("4.validation_model_evaluation/DCA_curves_allmodel_validation.pdf", dpi=300)

plt.show()

train_data = pd.read_csv("traindata.csv", encoding="GBK")

X_train_logist = train_data[['Smoking.History','aCCI.score', 'Preoperative.GO.SSS',

'SAS.score',

'Preoperative.K10', 'Preoperative.OFI.8']]

X_train_logist_const = sm.add_constant(X_train_logist)

y_train_logist = train_data['Outcome']

categorical_vars = train_data.iloc[:, 0:14].columns.tolist()

for var in categorical_vars:

train_data[var] = train_data[var].astype('category')

print(train_data.info())

train_data_ml = pd.read_csv("traindata.csv", encoding="GBK")

X_train = train_data_ml[['Smoking.History','aCCI.score', 'Preoperative.GO.SSS',

'SAS.score',

'Preoperative.K10', 'Preoperative.OFI.8']]

y_train = train_data_ml['Outcome']

y_train_pred_prob_logist = logist_model.predict(X_train_logist_const)

y_train_pred_logist = (y_train_pred_prob_logist >= 0.5).astype(int)

y_train_pred_prob_tree = tree_model.predict_proba(X_train)[:, 1]

y_train_pred_tree = (y_train_pred_prob_tree >= 0.5).astype(int)

y_train_pred_prob_rf = rf_model.predict_proba(X_train)[:, 1]

y_train_pred_rf = (y_train_pred_prob_rf >= 0.5).astype(int)

y_train_pred_prob_xgb = xgb_model.predict_proba(X_train)[:, 1]

y_train_pred_xgb = (y_train_pred_prob_xgb >= 0.5).astype(int)

y_train_pred_prob_lgb = lgb_model.predict_proba(X_train)[:, 1]

y_train_pred_lgb = (y_train_pred_prob_lgb >= 0.5).astype(int)

y_train_pred_prob_svm = svm_model.predict_proba(X_train)[:, 1]

y_train_pred_svm = (y_train_pred_prob_svm >= 0.5).astype(int)

y_train_pred_prob_ann = ann_model.predict_proba(X_train)[:, 1]

y_train_pred_ann = (y_train_pred_prob_ann >= 0.5).astype(int)

output_folder = "3.training_confusion_matrix"

cm_logist_train = confusion_matrix(y_train_logist, y_train_pred_logist)

CM_plot(cm_logist_train, "Logistic")

cm_tree_train = confusion_matrix(y_train, y_train_pred_tree)

CM_plot(cm_tree_train, "DecisionTree")

cm_rf_train = confusion_matrix(y_train, y_train_pred_rf)

CM_plot(cm_rf_train, "RandomForest")

cm_xgb_train = confusion_matrix(y_train, y_train_pred_xgb)

CM_plot(cm_xgb_train, "XGBoost")

cm_lgb_train = confusion_matrix(y_train, y_train_pred_lgb)

CM_plot(cm_lgb_train, "LightGBM")

cm_svm_train = confusion_matrix(y_train, y_train_pred_svm)

CM_plot(cm_svm_train, "SVM")

cm_ann_train = confusion_matrix(y_train, y_train_pred_ann)

CM_plot(cm_ann_train, "ANN")

accuracy_logist_train, precision_logist_train, sensitivity_logist_train, f1_logist_train, specificity_logist_train, kappa_logist_train, youden_logist_train, ppv_logist_train, npv_logist_train = calculate_acc_pre_sen_f1_spc(cm_logist_train, y_train_logist, y_train_pred_logist)

accuracy_tree_train, precision_tree_train, sensitivity_tree_train, f1_tree_train, specificity_tree_train, kappa_tree_train, youden_tree_train, ppv_tree_train, npv_tree_train = calculate_acc_pre_sen_f1_spc(cm_tree_train, y_train, y_train_pred_tree)

accuracy_rf_train, precision_rf_train, sensitivity_rf_train, f1_rf_train, specificity_rf_train, kappa_rf_train, youden_rf_train, ppv_rf_train, npv_rf_train = calculate_acc_pre_sen_f1_spc(cm_rf_train, y_train, y_train_pred_rf)

accuracy_xgb_train, precision_xgb_train, sensitivity_xgb_train, f1_xgb_train, specificity_xgb_train, kappa_xgb_train, youden_xgb_train, ppv_xgb_train, npv_xgb_train = calculate_acc_pre_sen_f1_spc(cm_xgb_train, y_train, y_train_pred_xgb)

accuracy_lgb_train, precision_lgb_train, sensitivity_lgb_train, f1_lgb_train, specificity_lgb_train, kappa_lgb_train, youden_lgb_train, ppv_lgb_train, npv_lgb_train = calculate_acc_pre_sen_f1_spc(cm_lgb_train, y_train, y_train_pred_lgb)

accuracy_svm_train, precision_svm_train, sensitivity_svm_train, f1_svm_train, specificity_svm_train, kappa_svm_train, youden_svm_train, ppv_svm_train, npv_svm_train = calculate_acc_pre_sen_f1_spc(cm_svm_train, y_train, y_train_pred_svm)

accuracy_ann_train, precision_ann_train, sensitivity_ann_train, f1_ann_train, specificity_ann_train, kappa_ann_train, youden_ann_train, ppv_ann_train, npv_ann_train = calculate_acc_pre_sen_f1_spc(cm_ann_train, y_train, y_train_pred_ann)

auc_value_logist_train, auc_ci_lower_logist_train, auc_ci_upper_logist_train = calculate_auc(y_train_logist, y_train_pred_prob_logist)

auc_value_tree_train, auc_ci_lower_tree_train, auc_ci_upper_tree_train = calculate_auc(y_train, y_train_pred_prob_tree)

auc_value_rf_train, auc_ci_lower_rf_train, auc_ci_upper_rf_train = calculate_auc(y_train, y_train_pred_prob_rf)

auc_value_xgb_train, auc_ci_lower_xgb_train, auc_ci_upper_xgb_train = calculate_auc(y_train, y_train_pred_prob_xgb)

auc_value_lgb_train, auc_ci_lower_lgb_train, auc_ci_upper_lgb_train = calculate_auc(y_train, y_train_pred_prob_lgb)

auc_value_svm_train, auc_ci_lower_svm_train, auc_ci_upper_svm_train = calculate_auc(y_train, y_train_pred_prob_svm)

auc_value_ann_train, auc_ci_lower_ann_train, auc_ci_upper_ann_train = calculate_auc(y_train, y_train_pred_prob_ann)

model_results_train = pd.DataFrame({

"Model": ["Logistic", "Decision Tree", "Random Forest", "XGBoost", "LightGBM", "SVM", "ANN"],

"AUC": [auc_value_logist_train, auc_value_tree_train, auc_value_rf_train, auc_value_xgb_train, auc_value_lgb_train, auc_value_svm_train, auc_value_ann_train],

"AUC 95% CI Lower": [auc_ci_lower_logist_train, auc_ci_lower_tree_train, auc_ci_lower_rf_train, auc_ci_lower_xgb_train, auc_ci_lower_lgb_train, auc_ci_lower_svm_train, auc_ci_lower_ann_train],

"AUC 95% CI Upper": [auc_ci_upper_logist_train, auc_ci_upper_tree_train, auc_ci_upper_rf_train, auc_ci_upper_xgb_train, auc_ci_upper_lgb_train, auc_ci_upper_svm_train, auc_ci_upper_ann_train],

"Accuracy": [accuracy_logist_train, accuracy_tree_train, accuracy_rf_train, accuracy_xgb_train, accuracy_lgb_train, accuracy_svm_train, accuracy_ann_train],

"Precision": [precision_logist_train, precision_tree_train, precision_rf_train, precision_xgb_train, precision_lgb_train, precision_svm_train, precision_ann_train],

"Sensitivity": [sensitivity_logist_train, sensitivity_tree_train, sensitivity_rf_train, sensitivity_xgb_train, sensitivity_lgb_train, sensitivity_svm_train, sensitivity_ann_train],

"Specificity": [specificity_logist_train, specificity_tree_train, specificity_rf_train, specificity_xgb_train, specificity_lgb_train, specificity_svm_train, specificity_ann_train],

"F1 Score": [f1_logist_train, f1_tree_train, f1_rf_train, f1_xgb_train, f1_lgb_train, f1_svm_train, f1_ann_train],

"Kappa": [kappa_logist_train, kappa_tree_train, kappa_rf_train, kappa_xgb_train, kappa_lgb_train, kappa_svm_train, kappa_ann_train],

"Youden's J": [youden_logist_train, youden_tree_train, youden_rf_train, youden_xgb_train, youden_lgb_train, youden_svm_train, youden_ann_train],

"PPV": [ppv_logist_train, ppv_tree_train, ppv_rf_train, ppv_xgb_train, ppv_lgb_train, ppv_svm_train, ppv_ann_train],

"NPV": [npv_logist_train, npv_tree_train, npv_rf_train, npv_xgb_train, npv_lgb_train, npv_svm_train, npv_ann_train]

})

model_results_train

model_results_train.to_csv("4.training_model_evaluation/model_performance_train.csv", index=False)

plt.figure(figsize=(8, 6))

models_train = {

"Logistic": (y_train_logist, y_train_pred_prob_logist),

"Decision Tree": (y_train, y_train_pred_prob_tree),

"Random Forest": (y_train, y_train_pred_prob_rf),

"XGBoost": (y_train, y_train_pred_prob_xgb),

"LightGBM": (y_train, y_train_pred_prob_lgb),

"SVM": (y_train, y_train_pred_prob_svm),

"ANN": (y_train, y_train_pred_prob_ann)

}

for model_name, (y_true, y_pred_prob) in models_train.items():

auc_value, auc_ci_lower, auc_ci_upper = calculate_auc(y_true, y_pred_prob)

fpr, tpr, _ = roc_curve(y_true, y_pred_prob)

plt.plot(fpr, tpr, label=f"{model_name} (AUC = {auc_value:.3f} [95% CI: {auc_ci_lower:.3f} - {auc_ci_upper:.3f}])")

plt.plot([0, 1], [0, 1], linestyle='--', color='gray')

plt.xlabel("1-Specificity")

plt.ylabel("Sensitivity")

plt.title("ROC Curve - Training Set Model Comparison")

plt.legend(loc='lower right')

plt.grid()

plt.savefig("4.training_model_evaluation/ROC_curves_allmodel_train.pdf", dpi=300)

plt.show()

plt.figure(figsize=(10, 8))

models_train = {

"Logistic": (y_train_logist, y_train_pred_prob_logist),

"Decision Tree": (y_train, y_train_pred_prob_tree),

"Random Forest": (y_train, y_train_pred_prob_rf),

"XGBoost": (y_train, y_train_pred_prob_xgb),

"LightGBM": (y_train, y_train_pred_prob_lgb),

"SVM": (y_train, y_train_pred_prob_svm),

"ANN": (y_train, y_train_pred_prob_ann)

}

for model_name, (y_true, y_pred_prob) in models_train.items():

prob_true, prob_pred = calibration_curve(y_true, y_pred_prob, n_bins=10)

brier_mean, brier_lower, brier_upper = brier_score_confidence_interval(y_true, y_pred_prob)

plt.plot(prob_pred, prob_true, marker='o', label=f'{model_name} (Brier Score: {brier_mean:.3f} [95% CI: {brier_lower:.3f} - {brier_upper:.3f}])')

plt.plot([0, 1], [0, 1], linestyle='--', color='gray', label='Perfect Calibration')

plt.xlabel("Predicted Probability")

plt.ylabel("True Probability")

plt.title("Calibration Curves - Training Set Model Comparison")

plt.legend(loc='lower right')

plt.grid()

plt.savefig("4.training_model_evaluation/Calibration_curves_allmodel_train.pdf", dpi=300)

plt.show()

plt.figure(figsize=(10, 8))

thresholds = np.linspace(0.01, 0.99, 100)

models_train = {

"Logistic": (y_train_logist, y_train_pred_prob_logist),

"Decision Tree": (y_train, y_train_pred_prob_tree),

"Random Forest": (y_train, y_train_pred_prob_rf),

"XGBoost": (y_train, y_train_pred_prob_xgb),

"LightGBM": (y_train, y_train_pred_prob_lgb),

"SVM": (y_train, y_train_pred_prob_svm),

"ANN": (y_train, y_train_pred_prob_ann)

}

for model_name, (y_true, y_pred_prob) in models_train.items():

net_benefit, net_benefit_alltrt, net_benefits_notrt = calculate_net_benefit(y_true, y_pred_prob, thresholds=thresholds)

plt.plot(thresholds, net_benefit, label=model_name)

plt.plot(thresholds, net_benefit_alltrt, linestyle="--", color="red", label="Treat All")

plt.plot(thresholds, net_benefits_notrt, linestyle="--", color="green", label="Treat None")

plt.xlabel("Threshold Probability")

plt.ylim(-0.2, np.nanmax(np.array(net_benefit)) + 0.1)

plt.ylabel("Net Benefit")

plt.title("Decision Curve Analysis - Training Set Model Comparison")

plt.legend(loc="lower right", fontsize=8)

plt.grid()

plt.savefig("4.training_model_evaluation/DCA_curves_allmodel_train.pdf", dpi=300)

plt.show()

test_data = pd.read_csv("traindata.csv", encoding="GBK")

X_test_logist = test_data[['Grade', 'Sepsis', 'Mechanical_ventilation', 'Age', 'GCS', 'SBP', 'Platelets', 'Glucose']]

X_test_logist_const = sm.add_constant(X_test_logist)

y_test_logist = test_data['Outcome']

categorical_vars = test_data.iloc[:, 0:20].columns.tolist()

for var in categorical_vars:

test_data[var] = test_data[var].astype('category')

print(test_data.info())

test_data_ml = pd.read_csv("traindata.csv", encoding="GBK")

X_test = test_data_ml[['Grade', 'Sepsis', 'Mechanical_ventilation', 'Age', 'GCS', 'SBP', 'Platelets', 'Glucose']]

y_test = test_data_ml['Outcome']

y_test_pred_prob_logist = logist_model.predict(X_test_logist_const)

y_test_pred_logist = (y_test_pred_prob_logist >= 0.5).astype(int)

y_test_pred_prob_tree = tree_model.predict_proba(X_test)[:, 1]

y_test_pred_tree = (y_test_pred_prob_tree >= 0.5).astype(int)

y_test_pred_prob_rf = rf_model.predict_proba(X_test)[:, 1]

y_test_pred_rf = (y_test_pred_prob_rf >= 0.5).astype(int)

y_test_pred_prob_xgb = xgb_model.predict_proba(X_test)[:, 1]

y_test_pred_xgb = (y_test_pred_prob_xgb >= 0.5).astype(int)

y_test_pred_prob_lgb = lgb_model.predict_proba(X_test)[:, 1]

y_test_pred_lgb = (y_test_pred_prob_lgb >= 0.5).astype(int)

y_test_pred_prob_svm = svm_model.predict_proba(X_test)[:, 1]

y_test_pred_svm = (y_test_pred_prob_svm >= 0.5).astype(int)

y_test_pred_prob_ann = ann_model.predict_proba(X_test)[:, 1]

y_test_pred_ann = (y_test_pred_prob_ann >= 0.5).astype(int)

output_folder = "3.external_test_confusion_matrix"

cm_logist_test = confusion_matrix(y_test_logist, y_test_pred_logist)

CM_plot(cm_logist_test, "Logistic")

cm_tree_test = confusion_matrix(y_test, y_test_pred_tree)

CM_plot(cm_tree_test, "DecisionTree")

cm_rf_test = confusion_matrix(y_test, y_test_pred_rf)

CM_plot(cm_rf_test, "RandomForest")

cm_xgb_test = confusion_matrix(y_test, y_test_pred_xgb)

CM_plot(cm_xgb_test, "XGBoost")

cm_lgb_test = confusion_matrix(y_test, y_test_pred_lgb)

CM_plot(cm_lgb_test, "LightGBM")

cm_svm_test = confusion_matrix(y_test, y_test_pred_svm)

CM_plot(cm_svm_test, "SVM")

cm_ann_test = confusion_matrix(y_test, y_test_pred_ann)

CM_plot(cm_ann_test, "ANN")

accuracy_logist_test, precision_logist_test, sensitivity_logist_test, f1_logist_test, specificity_logist_test, kappa_logist_test, youden_logist_test, ppv_logist_test, npv_logist_test = calculate_acc_pre_sen_f1_spc(cm_logist_test, y_test_logist, y_test_pred_logist)

accuracy_tree_test, precision_tree_test, sensitivity_tree_test, f1_tree_test, specificity_tree_test, kappa_tree_test, youden_tree_test, ppv_tree_test, npv_tree_test = calculate_acc_pre_sen_f1_spc(cm_tree_test, y_test, y_test_pred_tree)

accuracy_rf_test, precision_rf_test, sensitivity_rf_test, f1_rf_test, specificity_rf_test, kappa_rf_test, youden_rf_test, ppv_rf_test, npv_rf_test = calculate_acc_pre_sen_f1_spc(cm_rf_test, y_test, y_test_pred_rf)

accuracy_xgb_test, precision_xgb_test, sensitivity_xgb_test, f1_xgb_test, specificity_xgb_test, kappa_xgb_test, youden_xgb_test, ppv_xgb_test, npv_xgb_test = calculate_acc_pre_sen_f1_spc(cm_xgb_test, y_test, y_test_pred_xgb)

accuracy_lgb_test, precision_lgb_test, sensitivity_lgb_test, f1_lgb_test, specificity_lgb_test, kappa_lgb_test, youden_lgb_test, ppv_lgb_test, npv_lgb_test = calculate_acc_pre_sen_f1_spc(cm_lgb_test, y_test, y_test_pred_lgb)

accuracy_svm_test, precision_svm_test, sensitivity_svm_test, f1_svm_test, specificity_svm_test, kappa_svm_test, youden_svm_test, ppv_svm_test, npv_svm_test = calculate_acc_pre_sen_f1_spc(cm_svm_test, y_test, y_test_pred_svm)

accuracy_ann_test, precision_ann_test, sensitivity_ann_test, f1_ann_test, specificity_ann_test, kappa_ann_test, youden_ann_test, ppv_ann_test, npv_ann_test = calculate_acc_pre_sen_f1_spc(cm_ann_test, y_test, y_test_pred_ann)

auc_value_logist_test, auc_ci_lower_logist_test, auc_ci_upper_logist_test = calculate_auc(y_test_logist, y_test_pred_prob_logist)

auc_value_tree_test, auc_ci_lower_tree_test, auc_ci_upper_tree_test = calculate_auc(y_test, y_test_pred_prob_tree)

auc_value_rf_test, auc_ci_lower_rf_test, auc_ci_upper_rf_test = calculate_auc(y_test, y_test_pred_prob_rf)

auc_value_xgb_test, auc_ci_lower_xgb_test, auc_ci_upper_xgb_test = calculate_auc(y_test, y_test_pred_prob_xgb)

auc_value_lgb_test, auc_ci_lower_lgb_test, auc_ci_upper_lgb_test = calculate_auc(y_test, y_test_pred_prob_lgb)

auc_value_svm_test, auc_ci_lower_svm_test, auc_ci_upper_svm_test = calculate_auc(y_test, y_test_pred_prob_svm)

auc_value_ann_test, auc_ci_lower_ann_test, auc_ci_upper_ann_test = calculate_auc(y_test, y_test_pred_prob_ann)

model_results_test = pd.DataFrame({

"Model": ["Logistic", "Decision Tree", "Random Forest", "XGBoost", "LightGBM", "SVM", "ANN"],

"AUC": [auc_value_logist_test, auc_value_tree_test, auc_value_rf_test, auc_value_xgb_test, auc_value_lgb_test, auc_value_svm_test, auc_value_ann_test],

"AUC 95% CI Lower": [auc_ci_lower_logist_test, auc_ci_lower_tree_test, auc_ci_lower_rf_test, auc_ci_lower_xgb_test, auc_ci_lower_lgb_test, auc_ci_lower_svm_test, auc_ci_lower_ann_test],

"AUC 95% CI Upper": [auc_ci_upper_logist_test, auc_ci_upper_tree_test, auc_ci_upper_rf_test, auc_ci_upper_xgb_test, auc_ci_upper_lgb_test, auc_ci_upper_svm_test, auc_ci_upper_ann_test],

"Accuracy": [accuracy_logist_test, accuracy_tree_test, accuracy_rf_test, accuracy_xgb_test, accuracy_lgb_test, accuracy_svm_test, accuracy_ann_test],

"Precision": [precision_logist_test, precision_tree_test, precision_rf_test, precision_xgb_test, precision_lgb_test, precision_svm_test, precision_ann_test],

"Sensitivity": [sensitivity_logist_test, sensitivity_tree_test, sensitivity_rf_test, sensitivity_xgb_test, sensitivity_lgb_test, sensitivity_svm_test, sensitivity_ann_test],

"Specificity": [specificity_logist_test, specificity_tree_test, specificity_rf_test, specificity_xgb_test, specificity_lgb_test, specificity_svm_test, specificity_ann_test],

"F1 Score": [f1_logist_test, f1_tree_test, f1_rf_test, f1_xgb_test, f1_lgb_test, f1_svm_test, f1_ann_test],

"Kappa": [kappa_logist_test, kappa_tree_test, kappa_rf_test, kappa_xgb_test, kappa_lgb_test, kappa_svm_test, kappa_ann_test],

"Youden's J": [youden_logist_test, youden_tree_test, youden_rf_test, youden_xgb_test, youden_lgb_test, youden_svm_test, youden_ann_test],

"PPV": [ppv_logist_test, ppv_tree_test, ppv_rf_test, ppv_xgb_test, ppv_lgb_test, ppv_svm_test, ppv_ann_test],

"NPV": [npv_logist_test, npv_tree_test, npv_rf_test, npv_xgb_test, npv_lgb_test, npv_svm_test, npv_ann_test]

})

model_results_test

model_results_test.to_csv("4.external_test_model_evaluation/model_performance_test.csv", index=False)

plt.figure(figsize=(8, 6))

models_test = {

"Logistic": (y_test_logist, y_test_pred_prob_logist),

"Decision Tree": (y_test, y_test_pred_prob_tree),

"Random Forest": (y_test, y_test_pred_prob_rf),

"XGBoost": (y_test, y_test_pred_prob_xgb),

"LightGBM": (y_test, y_test_pred_prob_lgb),

"SVM": (y_test, y_test_pred_prob_svm),

"ANN": (y_test, y_test_pred_prob_ann)

}

for model_name, (y_true, y_pred_prob) in models_test.items():

auc_value, auc_ci_lower, auc_ci_upper = calculate_auc(y_true, y_pred_prob)

fpr, tpr, _ = roc_curve(y_true, y_pred_prob)

plt.plot(fpr, tpr, label=f"{model_name} (AUC = {auc_value:.3f} [95% CI: {auc_ci_lower:.3f} - {auc_ci_upper:.3f}])")

plt.plot([0, 1], [0, 1], linestyle='--', color='gray')

plt.xlabel("1-Specificity")

plt.ylabel("Sensitivity")

plt.title("ROC Curve - Test Set Model Comparison")

plt.legend(loc='lower right')

plt.grid()

plt.savefig("4.external_test_model_evaluation/ROC_curves_allmodel_test.pdf", dpi=300)

plt.show()

output_folder = "4.external_test_model_evaluation/single_model_evaluation"

ROC_plot(y_test_logist, y_test_pred_prob_logist, auc_value_logist_test, auc_ci_lower_logist_test, auc_ci_upper_logist_test, "Logistic")

ROC_plot(y_test, y_test_pred_prob_tree, auc_value_tree_test, auc_ci_lower_tree_test, auc_ci_upper_tree_test, "DecisionTree")

ROC_plot(y_test, y_test_pred_prob_rf, auc_value_rf_test, auc_ci_lower_rf_test, auc_ci_upper_rf_test, "RandomForest")

ROC_plot(y_test, y_test_pred_prob_xgb, auc_value_xgb_test, auc_ci_lower_xgb_test, auc_ci_upper_xgb_test, "XGBoost")

ROC_plot(y_test, y_test_pred_prob_lgb, auc_value_lgb_test, auc_ci_lower_lgb_test, auc_ci_upper_lgb_test, "LightGBM")

ROC_plot(y_test, y_test_pred_prob_svm, auc_value_svm_test, auc_ci_lower_svm_test, auc_ci_upper_svm_test, "SVM")

ROC_plot(y_test, y_test_pred_prob_ann, auc_value_ann_test, auc_ci_lower_ann_test, auc_ci_upper_ann_test, "ANN")

plt.figure(figsize=(10, 8))

models_test = {

"Logistic": (y_test_logist, y_test_pred_prob_logist),

"Decision Tree": (y_test, y_test_pred_prob_tree),

"Random Forest": (y_test, y_test_pred_prob_rf),

"XGBoost": (y_test, y_test_pred_prob_xgb),

"LightGBM": (y_test, y_test_pred_prob_lgb),

"SVM": (y_test, y_test_pred_prob_svm),

"ANN": (y_test, y_test_pred_prob_ann)

}

for model_name, (y_true, y_pred_prob) in models_test.items():

prob_true, prob_pred = calibration_curve(y_true, y_pred_prob, n_bins=10)

brier_mean, brier_lower, brier_upper = brier_score_confidence_interval(y_true, y_pred_prob)

plt.plot(prob_pred, prob_true, marker='o', label=f'{model_name} (Brier Score: {brier_mean:.3f} [95% CI: {brier_lower:.3f} - {brier_upper:.3f}])')

plt.plot([0, 1], [0, 1], linestyle='--', color='gray', label='Perfect Calibration')

plt.xlabel("Predicted Probability")

plt.ylabel("True Probability")

plt.title("Calibration Curves - Test Set Model Comparison")

plt.legend(loc='lower right')

plt.grid()

plt.savefig("4.external_test_model_evaluation/Calibration_curves_allmodel_test.pdf", dpi=300)

plt.show()

output_folder = "4.external_test_model_evaluation/single_model_evaluation"

CaliC_plot(y_test_logist, y_test_pred_prob_logist, "Logistic")

CaliC_plot(y_test, y_test_pred_prob_tree, "DecisionTree")

CaliC_plot(y_test, y_test_pred_prob_rf, "RandomForest")

CaliC_plot(y_test, y_test_pred_prob_xgb, "XGBoost")

CaliC_plot(y_test, y_test_pred_prob_lgb, "LightGBM")

CaliC_plot(y_test, y_test_pred_prob_svm, "SVM")

CaliC_plot(y_test, y_test_pred_prob_ann, "ANN")

plt.figure(figsize=(10, 8))

thresholds = np.linspace(0.01, 0.99, 100)

models_test = {

"Logistic": (y_test_logist, y_test_pred_prob_logist),

"Decision Tree": (y_test, y_test_pred_prob_tree),

"Random Forest": (y_test, y_test_pred_prob_rf),

"XGBoost": (y_test, y_test_pred_prob_xgb),

"LightGBM": (y_test, y_test_pred_prob_lgb),

"SVM": (y_test, y_test_pred_prob_svm),

"ANN": (y_test, y_test_pred_prob_ann)

}

for model_name, (y_true, y_pred_prob) in models_test.items():

net_benefit, net_benefit_alltrt, net_benefits_notrt = calculate_net_benefit(y_true, y_pred_prob, thresholds=thresholds)

plt.plot(thresholds, net_benefit, label=model_name)

plt.plot(thresholds, net_benefit_alltrt, linestyle="--", color="red", label="Treat All")

plt.plot(thresholds, net_benefits_notrt, linestyle="--", color="green", label="Treat None")

plt.xlabel("Threshold Probability")

plt.ylim(-0.2, np.nanmax(np.array(net_benefit)) + 0.1)

plt.ylabel("Net Benefit")

plt.title("Decision Curve Analysis - Test Set Model Comparison")

plt.legend(loc="lower right", fontsize=8)

plt.grid()

plt.savefig("4.external_test_model_evaluation/DCA_curves_allmodel_test.pdf", dpi=300)

plt.show()

output_folder = "4.external_test_model_evaluation/single_model_evaluation"

net_benefit_logist, net_benefit_alltrt_logist, net_benefits_notrt_logist = calculate_net_benefit(y_test_logist, y_test_pred_prob_logist)

DCA_plot(net_benefit_logist, net_benefit_alltrt_logist, net_benefits_notrt_logist, "Logistic")

net_benefit_tree, net_benefit_alltrt_tree, net_benefits_notrt_tree = calculate_net_benefit(y_test, y_test_pred_prob_tree)

DCA_plot(net_benefit_tree, net_benefit_alltrt_tree, net_benefits_notrt_tree, "DecisionTree")

net_benefit_rf, net_benefit_alltrt_rf, net_benefits_notrt_rf = calculate_net_benefit(y_test, y_test_pred_prob_rf)

DCA_plot(net_benefit_rf, net_benefit_alltrt_rf, net_benefits_notrt_rf, "RandomForest")

net_benefit_xgb, net_benefit_alltrt_xgb, net_benefits_notrt_xgb = calculate_net_benefit(y_test, y_test_pred_prob_xgb)

DCA_plot(net_benefit_xgb, net_benefit_alltrt_xgb, net_benefits_notrt_xgb, "XGBoost")

net_benefit_lgb, net_benefit_alltrt_lgb, net_benefits_notrt_lgb = calculate_net_benefit(y_test, y_test_pred_prob_lgb)

DCA_plot(net_benefit_lgb, net_benefit_alltrt_lgb, net_benefits_notrt_lgb, "LightGBM")

net_benefit_svm, net_benefit_alltrt_svm, net_benefits_notrt_svm = calculate_net_benefit(y_test, y_test_pred_prob_svm)

DCA_plot(net_benefit_svm, net_benefit_alltrt_svm, net_benefits_notrt_svm, "SVM")

net_benefit_ann, net_benefit_alltrt_ann, net_benefits_notrt_ann = calculate_net_benefit(y_test, y_test_pred_prob_ann)

DCA_plot(net_benefit_ann, net_benefit_alltrt_ann, net_benefits_notrt_ann, "ANN")

save_path = "5.SHAP_explanation/"

def shap_explain_model(model, X_train, X_val, model_name, n_interpret=150, obs_index=5):

explainer = shap.KernelExplainer(lambda X: model.predict_proba(X), X_train)

shap_values = explainer(X_val.iloc[:n_interpret, :])

plt.figure(figsize=(15, 12))

shap.plots.bar(shap_values[:, :, 1], max_display=15, show=False)

plt.savefig(f"{save_path}bar_{model_name}.pdf", dpi=200, bbox_inches='tight')

plt.close()

plt.figure(figsize=(15, 12))

shap.plots.beeswarm(shap_values[:, :, 1], max_display=15, show=False)

plt.savefig(f"{save_path}beeswarm_{model_name}.pdf", dpi=200, bbox_inches='tight')

plt.close()

shap_values_df = pd.DataFrame(shap_values[:, :, 1].values, columns=X_val.columns)

features = shap_values_df.columns.tolist()

num_features = len(features)

num_columns = 3

num_rows = (num_features + num_columns - 1) // num_columns

fig, axes = plt.subplots(num_rows, num_columns, figsize=(15, 5 * num_rows), dpi=200)

axes = axes.flatten()

for i in range(num_features):

feature = features[i]

ax = axes[i]

scatter = ax.scatter(X_val[feature][:n_interpret], shap_values_df[feature],

s=20, alpha=0.7, c=X_val[feature][:n_interpret], cmap='viridis')

ax.axhline(y=0, color='darkorange', linestyle='--', linewidth=1.5)

lowess_fit = lowess(shap_values_df[feature], X_val[feature][:n_interpret], frac=0.4)

ax.plot(lowess_fit[:, 0], lowess_fit[:, 1], color='darkred', linewidth=2.5, linestyle='-')

ax.set_xlabel(feature, fontsize=12)

ax.set_ylabel(f'SHAP value for\n{feature}', fontsize=12)

ax.spines['top'].set_visible(False)

ax.spines['right'].set_visible(False)

cbar = plt.colorbar(scatter, ax=ax, pad=0.02)

cbar.set_label('Feature value', fontsize=10)

for i in range(num_features, num_rows * num_columns):

axes[i].axis('off')

plt.tight_layout()

plt.savefig(f"{save_path}scatter_{model_name}.pdf", format='pdf', bbox_inches='tight')

plt.close()

print(f"All SHAP explanation images saved to {save_path}")

shap_explain_model(model=tree_model, X_train=X_train, X_val=X_val,

model_name="tree", n_interpret=150, obs_index=5)

shap_explain_model(model=rf_model, X_train=X_train, X_val=X_val,

model_name="rf", n_interpret=150, obs_index=5)

shap_explain_model(model=xgb_model, X_train=X_train, X_val=X_val,

model_name="xgb", n_interpret=150, obs_index=5)

shap_explain_model(model=lgb_model, X_train=X_train, X_val=X_val,

model_name="lgb", n_interpret=150, obs_index=5)

shap_explain_model(model=svm_model, X_train=X_train, X_val=X_val,

model_name="svm", n_interpret=150, obs_index=5)

shap_explain_model(model=ann_model, X_train=X_train, X_val=X_val,

model_name="ann", n_interpret=150, obs_index=5)

save_path = "5.SHAP_explanation/"

def shap_explain_model_force(model, X_train, X_val, model_name, n_interpret=150):

explainer = shap.KernelExplainer(lambda X: model.predict_proba(X), X_train)

shap_values = explainer(X_val.iloc[:n_interpret, :])

for class_index in [0, 1]:

plt.figure(figsize=(15, 12))

shap.plots.waterfall(shap_values[0][:, class_index], max_display=15, show=False)

plt.title(f"SHAP Waterfall Plot for Class {class_index}")

plt.savefig(f"{save_path}{model_name}_waterfall_1st_class{class_index}.pdf", format="pdf", bbox_inches='tight')

plt.show()

plt.close()

for class_index in [0, 1]:

force_plot = shap.plots.force(shap_values[2][:, class_index])

shap.save_html(f"{save_path}{model_name}_force_plot_3rd_class{class_index}.html", force_plot)

print(f"SHAP explanation images for {model_name} generated.")

shap_explain_model_force(model=tree_model, X_train=X_train, X_val=X_val,

model_name="tree", n_interpret=150)

shap_explain_model_force(model=rf_model, X_train=X_train, X_val=X_val,

model_name="rf", n_interpret=150)

shap_explain_model_force(model=xgb_model, X_train=X_train, X_val=X_val,

model_name="xgb", n_interpret=150)

shap_explain_model_force(model=lgb_model, X_train=X_train, X_val=X_val,

model_name="lgb", n_interpret=150)

shap_explain_model_force(model=svm_model, X_train=X_train, X_val=X_val,

model_name="svm", n_interpret=150)

shap_explain_model_force(model=ann_model, X_train=X_train, X_val=X_val,

model_name="ann", n_interpret=150)
